# Enabling robust blue circularly polarized organic afterglow through self-confining isolated chiral chromophore

**DOI:** 10.1038/s41467-024-47240-5

**Published:** 2024-04-09

**Authors:** Mingjian Zeng, Weiguang Wang, Shuman Zhang, Zhisheng Gao, Yingmeng Yan, Yitong Liu, Yulong Qi, Xin Yan, Wei Zhao, Xin Zhang, Ningning Guo, Huanhuan Li, Hui Li, Gaozhan Xie, Ye Tao, Runfeng Chen, Wei Huang

**Affiliations:** 1 State Key Laboratory of Organic Electronics and Information Displays & Institute of Advanced Materials (IAM), Nanjing University of Posts & Tele communications, Nanjing, China; 2 Songshan Lake Materials Laboratory, Dongguan, Guangdong China; 3 Frontiers Science Center for Flexible Electronics (FSCFE), MIIT Key Laboratory of Flexible Electronics (KLoFE), Northwestern Polytechnical University, Xi’an, Shanxi China

**Keywords:** Polymers, Organic molecules in materials science, Optical materials

## Abstract

Creating circularly polarized organic afterglow system with elevated triplet energy levels, suppressed non-radiative transitions, and effective chirality, which are three critical prerequisites for achieving blue circularly polarized afterglow, has posed a formidable challenge. Herein, a straightforward approach is unveiled to attain blue circularly polarized afterglow materials by covalently self-confining isolated chiral chromophore within polymer matrix. The formation of robust hydrogen bonds within the polymer matrix confers a distinctly isolated and stabilized molecular state of chiral chromophores, endowing a blue emission band at 414 nm, lifetime of 3.0 s, and luminescent dissymmetry factor of ~ 10^−2^. Utilizing the synergistic afterglow and chirality energy transfer, full-color circularly polarized afterglow systems are endowed by doping colorful fluorescent molecules into designed blue polymers, empowering versatile applications. This work paves the way for the streamlined design of blue circularly polarized afterglow materials, expanding the horizons of circularly polarized afterglow materials into various domains.

## Abstract (web summary)

The preparation of systems that give blue circularly polarised organic afterglow is desirable but challenging. Here, the authors confine a chiral chromophore within a polymer matrix to limit non-radiative transitions and give blue circularly polarised organic afterglow.

## Introduction

Blue circularly polarized luminescence (CPL), one of the three primary colors, is of paramount importance for the application in full-color stereoscopic displays, poly-chromatic data recording, and anti-counterfeiting as well as biological imaging and optical communications^1–14^. Varied organic blue CPL materials have been prepared including fluorescence^15–19^, metal-complex^20,21^ and thermally activated delayed fluorescence (TADF)^22,23^ materials, which have been applied in field-effect transistors^24^, organic light-emitting diodes^25^, and information storage^26^. Recently, circularly polarized organic afterglow (CPOA) has gained significant attention as a cutting-edge research field due to its exceptional photophysical properties for promising applications in various domains^27–33^. To design and develop CPOA materials, chiral chain engineering^34^, ionic co-crystal^35^, polymerization^36–38^, and multicomponent host-guest strategies^39–41^ have been proposed. Although remarkable achievements in enabling CPOA materials have been witnessed, developing blue CPOA materials with exceptionally prolonged lifetimes and effective chirality still remains a formidable challenge, let alone attaining adjustable CPOA emission colors.

**Fig. 1:**
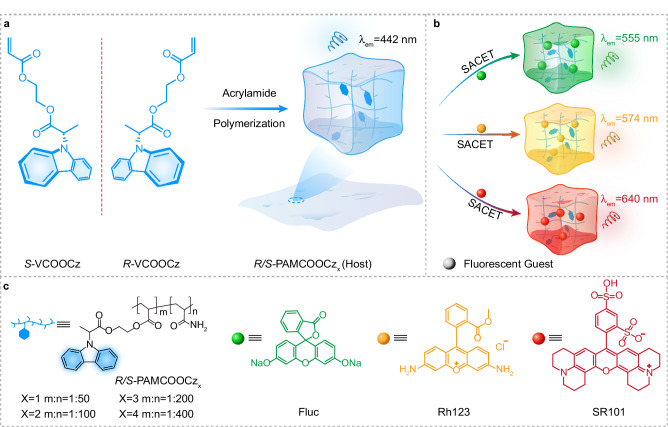
Construction of blue CPOA polymers for enabling full-color chiral afterglow emission. **a** Design of blue CPOA copolymer through covalent self-confinement of isolated chiral chromophores within a polymeric matrix. **b** Synergistic afterglow and chirality energy transfer (SACET) for achieving full-color CPOA polymers. **c** Molecular structures of PAMCOOCz_X_ (X = 1 ~ 4), fluorescent dyes fluorescein sodium (Fluc), rhodamine 123 (Rh123), and sulforodamine (SR101).

Three necessary prerequisites should be met to achieve CPOA emission. Firstly, the introduction of effective chirality into luminescent phosphor chromophores is essential^42^; secondly, it is imperative to enhance triplet exciton generation by expediting intersystem crossing (ISC) from singlet to triplet excited states^43^; lastly, the stabilization of the triplet excitons of phosphors through the construction of stable and stiff molecular environments for suppressing non-radiative decay pathways is of paramount need^44^. Proverbially, triplet excitons are easily exhausted through various non-radiative transition channels including triplet-triplet annihilation and luminescence quenching^45^. A possible approach to enhance CPOA emission is through chiral crystal engineering, which involves intermolecular locking and stacking to suppress non-radiative transitions of triplet excitons^46–49^. However, this tactic that leans upon ordered molecule packing may lead to the loss of triplet excitons through triplet-triplet annihilation and result in a spectrum shift towards longer wavelengths in the afterglow emission, making the development of long-lived blue CPOA challenging. Notably, compared to the aggregated state in molecular crystal^50^, the CPOA emission not only can experience a blue-shift when a single molecule is appropriately confined into a polymer matrix but also can empower the stabilization of triplet excitons for an ultralong lifetime. Based on these insights, we have proposed a strategy that involves the self-confinement of isolated chiral chromophores within a rigid polymer matrix to minimize non-radiative transitions for effectively boosting blue CPOA polymers (Fig. 1a). In this work, blue CPOA polymer demonstrates ultralong lifetimes of up to 3.0 s and maximum luminescent dissymmetry factor |*g*_lum_| value of 1.02 × 10^−2^. More excitingly, with the aid of synergistic afterglow and chirality energy transfer (SACET), full-color CPOA polymers with color tunability are achieved through doping commercially available water-soluble fluorescent materials, showing green, red, and even white CPOA emission with a lifetime of up to 2.1 s (Fig. 1b). These results not only provide a feasible way to develop blue CPOA materials but also signify the feasibility of the SACET strategy to construct full-color CPL materials.

## Results

### Material design and synthesis

As a proof of concept, a series of CPOA polymers *R/S*-PAMCOOCz_X_ (Fig. 1a) were synthesized through radical copolymerization. In this design, a pair of high triplet energy level enantiomers (Supplementary Figs. 1–15), *R/S*−2-((2-(9H-carbazol-9-yl) propa-noyl)oxy)thyl acrylatewith (*R/S*-VCOOCz)^51,52^, is chosen as the blue light-emitting monomer, which simultaneously exhibits good phosphorescent properties and chirality. Polyacrylamide (PAM), which has carbonyl and amino groups is chosen as a matrix because it not only can effectively promote ISC to generate the triplet excitons but also can form a strong hydrogen-bonding network to confine the blue chromophore for inhibited the non-radiative decay of triplet excitons.

### Photophysical properties of blue CPOA polymer

To ensure the chiral purity, the chiral resolution for *R*-VCOOCz and *S-*VCOOCz were performed. The enantiomeric excess values for *R*-VCOOCz and *S-*VCOOCz are calculated to be 99.9% and 98.1% (Supplementary Fig. 14). Moreover, the calculated circular dichroism (CD) spectra (Supplementary Fig. 15 and Supplementary Data 1) of *R*-VCOOCz and *S-*VCOOCz are consistent with the experimental spectra, which confirm the absolute configuration of *R*-VCOOCz and *S-*VCOOCz. Chiral polymers *R/S*-PAMCOOCz_X_ (X = 1 ~ 4) were synthesized through radical binary copolymerization using the self-designed central chiral monomer of *R/S*-VCOOCz and acrylamide (AM) (Supplementary Fig. 1) with molar feed ratio of 1:50 (*R/S*-PAMCOOCz_1_), 1:100 (*R/S*-PAMCOOCz_2_), 1:200 (*R/S*-PAMCOOCz_3_) and 1:400 (*R/S*-PAMCOOCz_4_). The structure characterizations of the target chiral monomer and polymers were confirmed by nuclear magnetic resonance spectroscopy, powder X-ray diffraction, and gel permeation chromatography (Supplementary Figs. 1–17 and Supplementary Table 1).

**Fig. 2:**
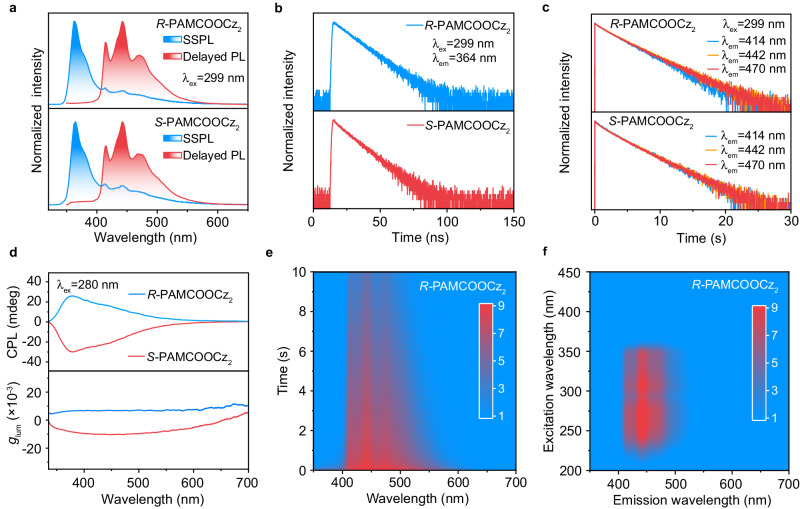
Photophysical properties of blue CPOA polymer *R/S*-PAMCOOCz_2_ films under ambient conditions. **a** Steady-state (blue line) (SSPL) and delayed (red line) PL spectra. **b, c** Fluorescence (**b**) and afterglow (**c**) decay profiles. **d** CPL (top panel) and corresponding *g*_lum_ curves (bottom panel). **e** Time-resolved emission spectrum. **f** Excitation-delayed PL emission spectrum.

*R/S*-PAMCOOCz_2_ shows carbazole-dominated absorption spectra in both solution and amorphous thin film, which exhibits π-π* absorption band at ~290 nm and *n*-π* absorption band near 330 nm (Supplementary Fig. 18), indicating that carbazole is the main absorption and chromophore unit^53^. Upon 254 nm UV light excitation, intense blue luminescence was recorded with emission peaks at 364 nm and lifetimes of 12.9 ns and 11.7 ns in *R-* and *S-*PAMCOOCz_2_ films (Fig. 2a, b and Supplementary Fig. 19). Excitingly, after the cease of excitation light, the obvious ultralong-lived blue afterglow could be observed by the naked eye under ambient conditions (Supplementary Fig. 19), showcasing emission peaks at 414, 442, and 470 nm (Fig. 2a) with lifetimes of 3.0, 3.1, and 3.1 s as well as 3.0, 3.1, and 3.0 s for *R-* and *S-*PAMCOOCz_2_ films (Fig. 2c and Supplementary Table 2), respectively. The CD spectra show mirror curves, which are consistent with the absorption spectra of *R/S*-PAMCOOCz_2_ films, suggesting the successful introduction of chirality into the blue afterglow *R/S*-PAMCOOCz_2_ polymers (Supplementary Fig. 20). CPL spectra reveal a strong and mirror signal (Fig. 2d, top panel) exhibiting main emission peaks at 364 nm with corresponding *g*_lum_ values of +6.4 × 10^−3^ and −7.8 × 10^−3^, and shoulder emission peaks at 442 nm with *g*_lum_ values of −1.02 × 10^−2^ and +6.8 × 10^−3^ for *R-* and *S-*PAMCOOCz_2_ films (Fig. 2d, bottom panel), respectively. These results indicate the achievement of a blue CPOA polymer.

To systematically investigate the effect of molar feed ratios on the blue CPOA emission, the chiral polymers with different molar feed ratios between *R/S*-VCOOCz and AM were constructed. Considering the quite similar photophysical properties of *R-* and *S-*PAMCOOCz_2_ films, *S-*PAMCOOCz_X_ polymers were selected as the model polymers (Supplementary Figs. 21 and 22) to perform the investigations. The afterglow intensities and CPL signals are firstly enhanced when the molar feed ratios of *S-*VCOOCz/AM rise from 1: 50 to 1: 100; however, with further increase the feed ratio of *S-*VCOOCz/AM from 1: 100 to 1: 200 and 1: 400, the afterglow intensities and CPL signals are gradually decreased. These results suggest that the rigidity of *R/S*-PAMCOOCz_2_ film suppresses non-radiative decay of triplet excitons, thus enabling elongated lifetime, improved afterglow intensity as well as enhanced CPL properties. It should be noted that, with further increase AM content, the hydrogen bonds in the corresponding polymeric films are largely enhanced, thus achieving identical lifetimes and non-radiaitve decay rates (Supplementary Table 3) in *S-*PAMCOOCz_X_ (X = 1 ~ 4) films; however, the concentration of chiral chromophores *S*-VCOOCz is decreased, leading to the largely decreased afterglow intensities and CPL signals. Considering the similar DC spectra (Supplementary Fig. 22), the higher CPL intensities endow higher *g*_lum_, thus empowering the *g*_lum_ values in the order of *S-*PAMCOOCz_2_ > *S-*PAMCOOCz_3_ > *S-*PAMCOOCz_4_ > *S-*PAMCOOCz_1_. Therefore, *R-* and *S-*PAMCOOCz_2_ films render the best CPOA attributes and their PLQY reached 28.6% and 24.7%, respectively (Supplementary Table 4). Notably, the copolymerization is much more effective than the physically blended polymer system of PAM and *R*-VCOOCz in endowing the CPOA emission (Supplementary Fig. 23).

In light of the excellent afterglow and CPL characters, *R/S*-PAMCOOCz_2_ films were chosen as the model polymers to investigate the blue CPOA properties. Time-resolved emission spectra (TRES) confirm that *R/S*-PAMCOOCz_2_ films have strong and stable afterglow luminescence (Fig. 2e and Supplementary Fig. 24). As shown in the excitation-delayed PL emission spectra (Fig. 2f and Supplementary Fig. 25), the blue CPOA could be effectively excited by UV light ranging from 210–360 nm with optimal excitation light at 299 nm. Interestingly, the excitation delayed PL spectra of emission peaks at 414, 442 and 470 nm in *R/S*-PAMCOOCz_2_ films are quite similar, indicating that these three emission peaks (414 nm, 442 nm, and 470 nm) originate from the same chromophore (Supplementary Fig. 26). To further investigate the luminescent source of *R/S*-PAMCOOCz_2_ films, low-temperature spectra of *R/S*-VCOOCz monomer, *R/S*-PAMCOOCz_2_, and PAM were performed (Supplementary Fig. 27). The low-temperature (77 K) delayed PL spectra of *R/S*-VCOOCz in dilute solution and polymethyl methacrylate doped film are consistent with these of the *R/S*-PAMCOOCz_2_ films at 77 K and room temperature, demonstrating the blue afterglow emission peaks at 414, 442, and 470 nm and high triplet energy level of 3.0 eV. Considering the combined results of the almost identical photophysical properties between *R/S*-PAMCOOCz_2_ and *R/S*-VCOOCz and a broader emission peak at ~420 nm of PAM films, the luminescent origination of blue CPOA polymer should be the isolated chiral *R/S*-VCOOCz chromophore. This self-confining isolated chromophore of *R/S*-VCOOCz monomer is further confirmed by the wide-angle X-ray scattering measurements (Supplementary Fig. 28), showing almost identical patterns to that of PAM film. Only broader scattering peaks at 1.54 A^−1^ arising from PAM film are observed^54^.

### Demonstration and investigation of SACET

**Fig. 3:**
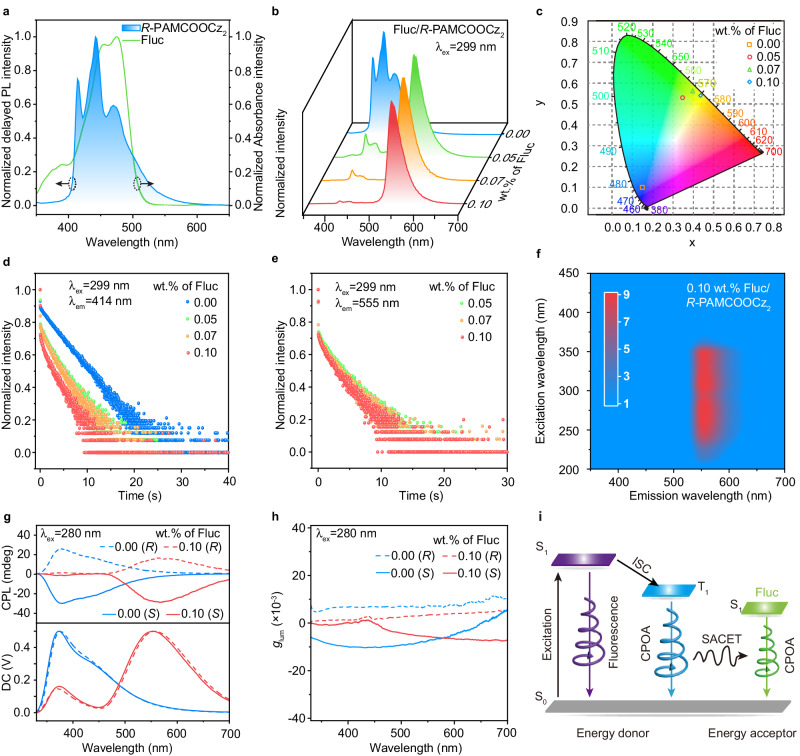
Demonstration and investigation of SACET. **a** Delayed PL spectra of *R-*PAMCOOCz_2_ film (delayed time, 10 ms) and absorption spectra of aqueous solutions (10^−5 ^mol/L) of Fluc. **b–e** Delayed PL spectra (**b**), Commission International de L’Eclairage (CIE) 1931 (**c**), lifetime decay profiles at 414 nm (**d**) and 555 nm (**e**) of Fluc/*R-*PAMCOOCz_2_ at different concen-trations. **f** Excitation-delayed PL emission spectrum of 0.1 wt.% Fluc/*R-*PAMCOOCz_2_ film. **g, h** CPL spectra (**g**) and *g*_lum_ values (**h**) of Fluc/*R-*PAMCOOCz_2_ films with doping concentrations of 0.0 wt.% and 0.1 wt.%. **i** Schematic diagram of the SACET mechanism.

Benefitting from the blue chiral afterglow emission with ultralong lifetime of 3.0 s and chirality with maximum *g*_lum_ of 1.02 × 10^−2^ as well as water-solubility, wide afterglow emission spectrum ranging from 400–550 nm and acceptable oscillator strength, *R/S*-PAMCOOCz_2_ could be an ideal host platform to on-demand construct full-color CPOA polymers with robust and tunable afterglow emission^55^. Therefore, the commercialized water-soluble fluorescent dyes of fluorescein sodium (Fluc, λ_abs_ = 400–480 nm), rhodamine 123 (Rh123, λ_abs_ = 450–520 nm), and sulfo-rhodamine (SR101, λ_abs_ = 520–640 nm) were selected as guest to develop the full-color CPOA polymers due to the large spectra overlap between the afterglow spectra of energy donor *R/S*-PAMCOOCz_2_ (host) and the absorption spectra of energy acceptor (fluorescent guest)^56–58^, respectively. The well-overlapped spectra could maintain effective SACET from host to guest (Fig. 3a and Supplementary Fig. 29). Additionally, both the host and guest are water-soluble, leading to good compatibility for the physically blended host-guest system to shorten the distance between the donor and acceptors for further enhancing ET efficiency. Experimentally, to confirm the SACET, Fluc/*R-*PAMCOOCz_2_ films with different Fluc weight concentrations were fabricated by mixing and evaporating the aqueous solution of Fluc and *R-*PAMCOOCz_2_. As shown in Fig. 3b, besides the blue emission from *R-*PAMCOOCz_2_, newly emerged luminescent peaks are found in both SSPL and delayed PL spectra in Fluc/*R-*PAMCOOCz_2_ film. Compared to the emission peak of Fluc in aqueous solution and an inert poly (vinyl alcohol) doped film (Supplementary Fig. 30), the newly emerged luminescence peak at 555 nm in the SSPL and delayed PL spectra of the Fluc/*R-*PAMCOOCz_2_ film should be from Fluc; and with rising Fluc concentrations, the intensities of emission peak at 550–650 nm are gradually increased while the emission from *R-*PAMCOOCz_2_ is decreased, signifying the plausible occurrence of ET from *R-*PAMCOOCz_2_ to Fluc. Compared to the SSPL emission (Supplementary Fig. 31a), the afterglow emission from *R-*PAMCOOCz_2_ almost disappeared in Fluc/*R-*PAMCOOCz_2_ film when the doping concentration of Fluc increased to 0.1 wt.%, demonstrating effective ET for achieving tunable afterglow emission. Eventually, the afterglow achieves a shift from blue to yellow-green (Fig. 3c).

To investigate the specific ET mechanism in depth, the lifetimes of the Fluc/*R-*PAMCOOCz_2_ films were analyzed. Upon increasing Fluc concentrations from 0 to 0.1 wt.%, the lifetimes are decreased from 12.9 ns to 9.4 ns for fluorescence emission peaks at 364 nm and from 3.0 s to 2.0 s for afterglow emission peaks at 414 nm (Supplementary Fig. 31b and Fig. 3d), respectively. These results verify that the non-radiative ET process should be responsible for this ultralong afterglow emission from Fluc. The afterglow lifetimes of long-lived emission peaks at 555 nm are over 1.8 s (Fig. 3e and Supplementary Table 5). According to the measured amplitude averaged lifetime (τ_amp_) of Fluc/*R-*PAMCOOCz_2_ films at the emission peaks at 364, and 414 nm, the fluorescence and afterglow ET efficiencies are calculated to be 27.1% and 64.3%, respectively (Supplementary Table 6). Compared to the fluorescence ET efficiency, much-enhanced afterglow ET efficiency should be due to its larger spectra overlap between the afterglow emission of *R-*PAMCOOCz_2_ and the absorption spectrum of Fluc. Moreover, the TRES of Fluc/*R-*PAMCOOCz_2_ film shows continuous and pronounced luminescence with elongating the delayed time (Supplementary Fig. 32), suggesting the stability of the sensitized ultralong afterglow luminescence from Fluc, demonstrating that the ET strategy should be an alternative way to modulate the afterglow color^59^. With increase Fluc concentrations, the photoluminescence quantum efficiencies of the Fluc/*R-*PAMCOOCz_2_ system are also increased from 23.3% to 28.1% (Supplementary Table 4). The excitation-delayed PL spectra of *R-*PAMCOOCz_2_ and 0.1 wt.% Fluc/*R-*PAMCOOCz_2_ (Fig. 3f) are almost identical, showing quite similar excitation-delayed PL spectra with effective excitation wavelength from 208 nm to 362 nm. In contrast, no afterglow luminescence can be observed when the excitation wavelength ranges from 400 nm to 450 nm where Fluc exhibits strong absorption abilities, indicating that the ultralong lifetime triplet excitons of Fluc/*R-*PAMCOOCz_2_ films should be attributed to the afterglow ET from *R-*PAMCOOCz_2_ to Fluc rather than the direct excitation of Fluc. The Fluc/*S-*PAMCOOC_2_ systems endow similar spectra variations when modulated the doping concentrations of Fluc.

Since the Fluc/*R-*PAMCOOCz_2_ and Fluc/*S-*PAMCOOCz_2_ films empower effectual fluorescence and afterglow ET process, their chirality energy transfer properties were subsequently investigated. In comparison with the *R-*PAMCOOCz_2_ film, a new peak at 555 nm is observed after excitation by a 280 nm light. With increased Fluc concentrations in Fluc/*R-*PAMCOOCz_2_ film, the enhanced CPL signals originating from 555 nm are found while the CPL signals of *R-*PAMCOOCz_2_ film at the regions of 330–450 nm are gradually decreased (Fig. 3g and Supplementary Fig. 33a). After the aiding of 0.1 wt.% Fluc, the CPL signal of *R/S*-PAMCOOCz_2_ almost disappears, and only a strong CPL signal at 555 nm is retained with *g*_lum_ values of + 3.4 × 10^−3^ and − 5.7 × 10^−^^3^, respectively (Fig. 3h, i and Supplementary Fig. 33b). Namely, the chirality of *R/S*-PAMCOOCz_2_ is successfully transferred to Fluc. These results agree well with the variation of SSPL emission of Fluc/*R*-PAMCOOCz_2_ films (Supplementary Fig. 31a). Notably, the CD spectra (Supplementary Fig. 34) of Fluc doped *R/S*-PAMCOOCz_2_ films are similar to the corresponding CD spectra of *R/S*-PAMCOOCz_2_ films (Supplementary Fig. 20) and the use of strong absorption peak at 460 nm of Fluc as the excitation wavelength (Fig. 3a) could not trigger the CPL emission of Fluc/*S-*PAMCOOCz_2_ (Supplementary Fig. 35), confirming that the chirality of Fluc doped systems originates from the chiral *R/S*-PAMCOOCz_2_ hosts^60–62^.

### Enabling multicolor CPOA polymers

**Fig. 4:**
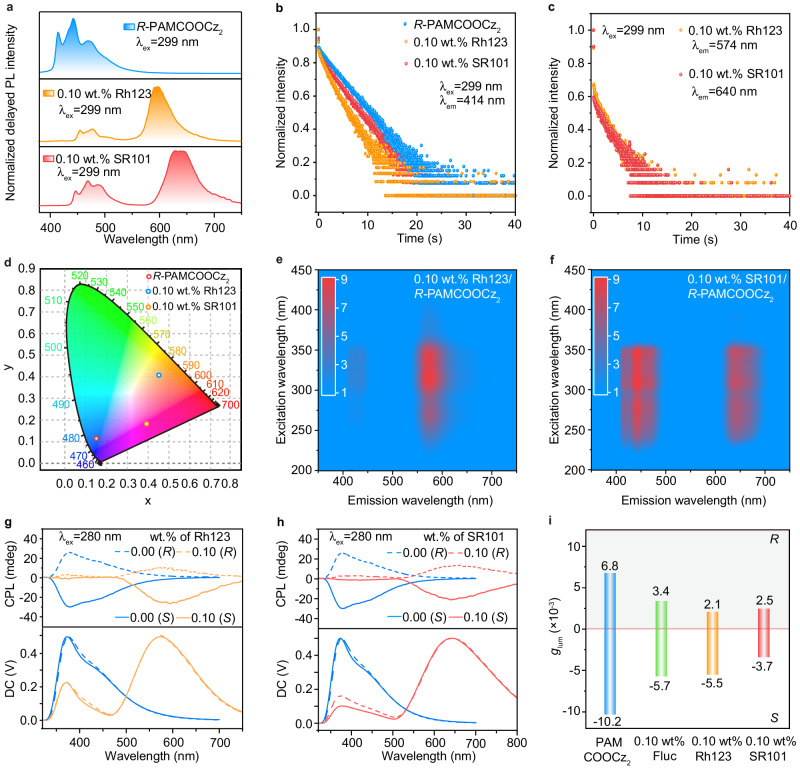
Enabling multicolor CPOA polymers. **a–d** Delayed PL spectra (**a**), lifetime decay profiles (**b, c**) and CIE 1931 (**d**) of *R-*PAMCOOCz_2_, 0.1 wt.% Rh123/*R-*PAMCOOCz_2_ and 0.1 wt.% SR101/*R-*PAMCOOCz_2_. **e, f** Excitation-delayed PL emission spectrum of 0.1 wt.% Rh123/*R-*PAMCOOCz_2_ (**e**) and 0.1 wt.% SR101/*R-*PAMCOOCz_2_ (**f**) films. **g, h** CPL spectra of Rh123/*R-*PAMCOOCz_2_ (**g**) and SR101/*R-*PAMCOOCz_2_ (**h**) films with doping concentration of 0.0 wt.% and 0.1 wt.%. **i** *g*_lum_ values of emission peaks at 440, 555, 574 and 640 nm for varied fluorescent guests doped *R/S*-PAMCOOCz_2_ films with doping concentrations of 0.0 wt.% and 0.1 wt.% (top panel is *R-*configuration and bottom panel is *S-*configuration).

To further demonstrate the university of the SACET to obtain wide-range color tenability^16^, the achiral Rh123 and SR101 were chosen as fluorescence guests to prepare thin films since their absorption spectra endow favorable spectra overlaps with afterglow emission of *R/S*-PAMCOOCz_2_ films. As anticipated, orange and red chiral afterglow emission peaked at 574 nm and 640 nm, corresponding to the luminescence peaks of Rh123 and SR101, are observed in 0.1 wt.% Rh123 and SR101 doped *R-*PAMCOOCz_2_ films, respectively (Fig. 4a and Supplementary Fig. 36). Compared to pure *R-*PAMCOOCz_2_ film, significantly reduced delayed PL intensities and lifetimes are found in the emission peaks at 414 nm in Rh123 and SR101 doped films (Fig. 4b), proving the occurrence of afterglow ET. This ET can be further verified by excitation-delayed PL spectra and emission mapping, which show quite similar excitation wavelength to that of the *R-*PAMCOOCz_2_ host (Fig. 4e, f and Supplementary Fig. 37). Since the doping concentration of the guest is vital to enable multicolor CPOA, the SR101 doped *R-*PAMCOOCz_2_ films with the weight concentrations of 0.05 wt.%, 0.1 wt.%, and 0.2 wt.% were prepared; As shown in Supplementary Fig. 38, 0.1 wt.% SR101/*R-*PAMCOOCz_2_ film demonstrates much enhanced SSPL and afterglow emission that originates from SR101, suggesting that the optimal doping concentration is 0.1 wt.%. Moreover, 0.1 wt.% Rh123 and SR101 doped *R-*PAMCOOCz_2_ films obtain strong and stable long afterglow luminescence (Supplementary Fig. 32) and show ultralong lifetimes of 1.9 and 2.2 s (Fig. 4c, d and Supplementary Table 7).

Expectedly, when 0.1 wt.% Rh123 and SR101 were doped into *R/S*-PAMCOOCz_2_ films, the CPL signal originated from *R/S*-PAMCOOCz_2_ is largely decreased, while newly emerged emission bands derived from Rh123 (574 nm) and SR101 (640 nm) are observed (Fig. 4g, h), respectively. The maximum *g*_lum_ of +2.1 × 10^−^^3^ and −5.5 × 10^−^^3^, + 2.5 × 10^−^^3^, and −3.7 × 10^−^^3^ for 0.1 wt.% Rh123 and SR101 doped *R/S*-PAMCOOCz_2_ films could be also recorded (Fig. 4i and Supplementary Fig. 39), figuring out the effective chirality transfer from *R/S*-PAMCOOCz_2_ to fluorescent guests. Compared to *R/S*-PAMCOOCz_2_ and Fluc doped *R/S*-PAMCOOCz_2_ films, the reduced CPL signals of 0.1 wt.% Rh123 and SR101 doped *R/S*-PAMCOOCz_2_ films should be due to their decreased energy transfer efficiencies (Supplementary Table 6)^63^. To further verify the vital role of SACET to achieve multicolor CPOA system, the direct excitation of the achiral guest SR101 in 0.1 wt.% SR101/*R-*PAMCOOCz_2_ films using the corresponding maximum absorption band at 550 nm as excitation light was performed. No obvious CPL signals are detected (Supplementary Fig. 40), testifying that the chiral characteristics should be derived from blue *R/S*-PAMCOOCz_2_ polymer. More excitingly, chiral white light emission can be achieved in Rh123/*R-*PAMCOOCz_2_ films by carefully regulating the doping concentration of Rh123 (Supplementary Fig. 41).

### Potential applications of CPOA materials

**Fig. 5:**
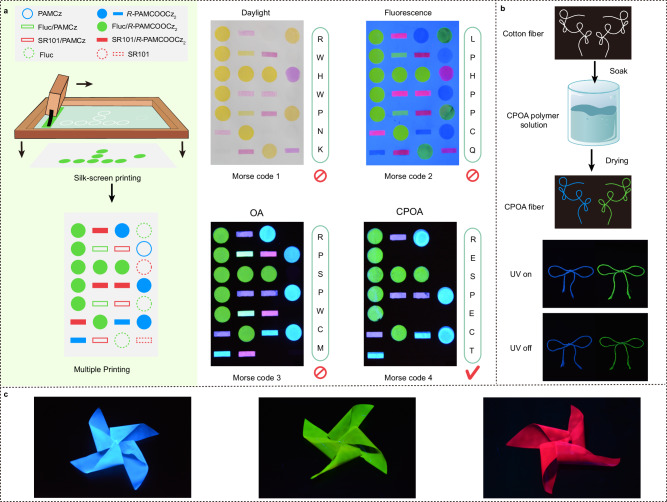
Potential applications of CPOA materials. **a** Fabrication procedure of multiplex Morse Code by screen-printing and corresponding photographs of varied Morse Code under daylight, under 254 nm UV light excitation and after turning off 254 nm UV light excitation. **b** Preparation procedures and an illustration of CPOA fibers and corresponding photographs of CPOA fibers under and after turning off 254 nm UV light excitation. **c** Blue (left), yellow-green (middle), and red (right) three-dimensional CPOA objects.

Considering CPOA materials capable of ultralong lifetime, full-color tunability, and easy water processing ability, their applications in multiplex information encryption, functionalization fibers and three-dimensional objects were explored. As shown in Fig. 5a, various luminescent materials including chiral *R-*PAMCOOCz_2_ (blue), Fluc/*R-*PAMCOOCz_2_ (yellow-green), and SR101/*R-*PAMCOOCz_2_ (red), and achiral PAMCz (blue), Fluc (yellow-green), Fluc/PAMCz (yellow-green), SR101 (red) and SR101/PAMCz (red) were selected as the water-soluble anticounterfeiting inks (Supplementary Fig. 42). Multiplex Morse Codes with a three-dimensional encrypted features of CPL and color, long-life had been prepared using screen-printing technology (Supplementary Fig. 43). Under daylight, due to the colorless of blue inks, the false Morse Code 1 RWHWPNK with light-yellow and pink color can be obtained. Upon irradiation by a 254 nm UV lamp, all emission colors can be observed and the false fluorescence Morse Code 2 LPHPPCQ can be recorded. After withdrawing the UV lamp, the false OA Morse Code 3 RPSPWCM with red, green, and blue colors appeared as the Fluc and SR101 fluorescence disappeared. In contrast, with the aid of CPL analysis, the true three-dimensional encrypted CPOA Morse Code 4 showing the information of RESPECT is finally captured (Supplementary Fig. 44). Meanwhile, CPOA functionalized fibers could be easily prepared by soaking the commercial fiber into the CPOA polymer aqueous solution and then drying in an oven at 50 °C, rendering varied shapes and the tunable afterglow of blue (*R-*PAMCOOCz_2_) and green (Fluc/*R-*PAMCOOCz_2_) emission colors (Fig. 5b). Interestingly, colorful three-dimensional objects (Fig. 5c) emitting blue (*R-*PAMCOOCz_2_, left), yellow-green (0.1 wt.% Fluc/*R-*PAMCOOCz_2_, middle), and red (0.1 wt.% SR101/*R-*PAMCOOCz_2_) CPOA emissions can be also constructed, reflecting their great potential applications in flexible wearable electronics.

## Discussion

In summary, we have successfully proposed an efficient self-confinement method for the construction of blue CPOA polymers. This tactic depends on simultaneously confining the isolated chiral chromophores into a rigid and water-soluble polymer matrix for the stabilization of triplet excitons to enable highly efficient CPOA emission. The resultant water-soluble CPOA polymers exhibit an ultralong lifetime of up to 3.0 s, a |*g*_lum_| of 1.02 × 10^−2^. Benefiting from afterglow and chirality properties, a series of full-color CPOA systems are prepared by physically blending water-soluble non-chiral fluorescent dyes into blue CPOA polymers through SACET. Employing the full-color tunability CPOA features, multilevel information encryption, functional fibers, and three-dimensional display objects have been fabricated. This study not only guides the design and synthesis of blue CPOA materials, but also opens a map to on-demand modulate the CPOA emission colors for varied applications.

## Methods

### Materials

All reagents and solvents were purchased from Nanjing Chemical Reagent Co. and Energy Chemical. Unless otherwise specified, these reagents and solvents were used without further purification. The purities for all purchased materials are described below: carbazole (99%), methy (R)-2-chloropropionate (98%), methy (S)-2-chloropropionate (97%), 2-hydroxyethyl acrylate (99%), acrylamide (99.9%), fluorescein sodium (99.7%), rhodamine 123 (98%), sulfo-rhodamine (95%). And 2,2’-azobis(2-methylpropionitrile) (98%) was used after three times recrystallization.

### General procedure of radical polymerization

In an argon atmosphere, 0.01 equivalent (eq) of 2,2’-azobis(2-methylpropionitrile) (AIBN) and 1.0 eq of vinyl derivative were dissolved in 25 mL freshly distilled tetrahydrofuran (THF) under ice water. After the solid was completely dissolved, the mixture was gradually heated to 55 °C and stirred for 16 h. After the reaction, the mixture was added dropwise to 200 mL methanol to precipitate polymeric materials, then the crude product was filtered, followed by washing with petroleum ether and dichloromethane, acetone in sequence. Then the solid was dissolved in deionized water and dialyzed by a dialysis tube (molecular weight cut-off = 1000) for 72 h.

### *S-*PAMCOOCz_1_

Following the general procedure of radical polymerization using *S*-VCOOCz (0.337 g, 1.0 mmol, 1.00 eq), acrylamide (AM, 3.55 g, 50.0 mmol, 50 eq), and appropriate amount of AIBN (0.0836 g, 0.51 mmol, 0.51 eq) in 25 mL freshly distilled THF to afford 3.31 g white powder polymer with a yield of 85.2%. Mn = 16817 Da; Mw = 25661 Da; PDI = 1.53.

### *S-*PAMCOOCz_2_

Following the general procedure of radical polymerization using *S*-VCOOCz (0.169 g, 0.5 mmol, 1.00 eq), acrylamide (3.55 g, 50.0 mmol, 100 eq), and appropriate amount of AIBN (0.0828 g, 0.505 mmol, 1.01 eq) in 25 mL freshly distilled THF to afford 3.51 g white powder polymer with a yield of 94.5%. Mn = 23603 Da; Mw = 38866 Da; PDI = 1.65.

### *R*-PAMCOOCz_2_

Following the general procedure of radical polymerization using *R*-VCOOCz (0.169 g, 0.5 mmol, 1.00 eq), acrylamide (3.55 g, 50.0 mmol, 100 eq), and appropriate amount of AIBN (0.0828 g, 0.505 mmol, 1.01 eq) in 25 mL freshly distilled THF to afford 3.56 g white powder polymer with a yield of 95.8%. Mn = 22006 Da; Mw = 38117 Da; PDI = 1.73.

### *S*-PAMCOOCz_3_

Following the general procedure of radical polymerization using *S*-VCOOCz (0.0843 g, 0.25 mmol, 1.00 eq), acrylamide (3.55 g, 50.0 mmol, 200 eq), and appropriate amount of AIBN (0.0824 g, 0.5025 mmol, 2.01 eq) in 25 mL freshly distilled THF to afford 3.51 g white powder polymer with a yield of 96.6%. Mn = 25102 Da; Mw = 41739 Da; PDI = 1.66.

### *S*-PAMCOOCz_4_

Following the general procedure of radical polymerization using *S*-VCOOCz (0.0421 g, 0.125 mmol, 1.00 eq), acrylamide (3.55 g, 50.0 mmol, 400 eq), and appropriate amount of AIBN (0.0822 g, 0.5013 mmol, 4.01 eq) in 25 mL freshly distilled THF to afford 3.50 g white powder polymer with a yield of 97.4%. Mn = 29095 Da; Mw = 46679 Da; PDI = 1.60.

### Preparation of full-color polymer films

0.5 g of polymer powder and a certain amount of organic fluorescent dyes were dissolved in deionized water (10 mL) followed by vigorous sonication for 10 min under ambient conditions; then the solution was poured into a flask and stirred at 60 °C for 1 h to obtain a completely transparent polymer solution; finally the mixed solution was placed in a petri dish and dried in an oven at 70 °C overnight to fabricate transparent polymer films for subsequent photophysical and morphological characterizations.

### Supplementary information

Supplementary Information

Peer Review File

Description of Additional Supplementary Files

Supplementary Data 1

### Source data

Source Data

## Data Availability

The data that support the plots within this paper and other findings of this study are available from the corresponding author on request. Source data are provided in this paper.
