# Supplementary material for: Enabling robust blue circularly polarized organic afterglow through self-confining isolated chiral chromophore
Source: Nat Commun. 2024 Apr 9;15:3053. doi: 10.1038/s41467-024-47240-5 (PMC11004163; doi:10.1038/s41467-024-47240-5)
Supplement: Supplementary file 1 — Supplementary Information [file 41467_2024_47240_MOESM1_ESM.pdf]

Supplementary Information

Enabling robust blue circularly polarized organic afterglow through self-confining isolated chromophore

Mingjian Zeng[a], Weiguang Wang[a], Shuman Zhang[a], Zhisheng Gao[a], Yingmeng Yan[a], Yitong Liu[a], Yulong Qi[a], Xin Yan[a], Wei Zhao[a], Xin Zhang[a], Ningning Guo[a], Huanhuan Li[a], Hui Li[a], Gaozhan Xie[a], Ye Tao*[a, b], Runfeng Chen*[a] and Wei Huang*[a, c]

[a] State Key Laboratory of Organic Electronics and Information Displays & Institute of Advanced Materials (IAM), Nanjing University of Posts & Tele communications, 210023 Nanjing, China.

[b] Songshan Lake Materials Laboratory, Dongguan, Guangdong, 523808 China

[c] Frontiers Science Center for Flexible Electronics (FSCFE), MIIT Key Laboratory of Flexible Electronics (KLoFE), Northwestern Polytechnical University, Xi'an 710072, Shanxi, China.

E-mail: iamytao@njupt.edu.cn; iamrfchen@njupt.edu.cn; iamwhuang@njupt.edu.cn.

Supplementary Methods

Measurements: The nuclear magnetic resonance (NMR) spectra were measured on a Bruker Ultra Shield Plus 400 MHz NMR instrument using tetramethylsilane (TMS) as the reference standard and deuterated chloroform ($CDCl_3$), deuterated dimethyl sulfoxide (DMSO-d_6), and deuterated water (D_2O) as solvents. The chemical shifts (δ) are in ppm, the coupling constant (J) is in Hz, and the fractional peaks used are identified as: s (single peak), d (double peak), and m (multiple peak). The molecular weights of the polymers were characterized by aqueous gel permeation chromatography (GPC) with a mobile phase of 0.1 mol/L sodium nitrate and a flow rate of 0.7 mL min^{-1}. Powder X-ray diffraction (XRD) was measured on a Bruker D8 Advance diffractometer with Cu Kα (λ = 1.5418 Å) radiation at room temperature. Ultraviolet/Visible (UV/Vis) absorption spectra were characterized on a Jasco V-750 spectrophotometer. Steady-state photoluminescence (SSPL) spectra, delayed PL spectra (delay time 10 ms), fluorescence/phosphorescence lifetime decay curves, time-resolved emission spectra (TRES) and absolute photoluminescence quantum efficiency (PLQY) were conducted on an Edinburgh FLS980 spectrometer. For the fluorescence lifetime test, a picosecond pulsed laser (EPLED-295, wavelength: 295 nm, pulse width: 833.7 ps) was used; for the PLQY test, an integrating sphere was used in the Edinburgh FLS980 instruments, and the wavelength-dependent sensitivity of the detector has been calibrated automatically by Edinburgh instruments during PLQY measurement. Excitation-delayed PL spectra were characterized with a Hitachi F-4700 spectrometer (delay time: 25 ms). Photographs were taken with a Nikon D7100 camera. Chiral high performance liquid chromatography (HPLC) measurements were performed on Shimadzu LC-20AT using CHIRALCEL OJ-H column and methanol as mobile phase. The circular dichroism (CD) spectra were measured on a Chirascan V100 CD spectrometer. The transmittance mode (180 degrees) with 'Low' sensitivity was used to perform the CD spectra measurement. The scan speed was set as 200 nm/min with 1 nm resolution and a response time of 1.0 s. Circularly polarized luminescence (CPL) spectra were measured with a JASCO CPL-300 spectrometer. Wide-angle X-ray scattering patterns were performed using the Xeuss 2.0 (Xenocs, France) with an incident X-ray Cu-Kα beam (λ = 1.54189 Å).

The lifetimes (τ) of the luminescence were obtained by fitting the decay curve with a multi-exponential decay function of

$$I(t) = \sum_i B_i e^{-\frac{t}{\tau_i}} \quad (1)$$

Where B_i and τ_i represent the amplitudes and lifetimes of the individual components for multi-exponential decay profiles, respectively.

The average lifetime was calculated by the function of

$$\tau_{ave} = \sum_i \varphi_i \tau_i \quad (2)$$

where φ_i is the amplitude fraction.

To get the intensity-averaged lifetime (τ_{int}), the φ_i^{int} is defined by the function of

$$\varphi_i^{int} = \frac{B_i \tau_i}{\sum_i B_i \tau_i} \times 100\% \quad (3)$$

τ_{int} is achieved by the function of

$$\tau_{int} = \sum_i \varphi_i^{int} \tau_i \quad (4)$$

To get the amplitude averaged lifetime (τ_{amp}) which was used for the analyses of SACET process, the φ_i^{amp} is defined by the function of:

$$\varphi_i^{amp} = \frac{B_i}{\sum_i B_i} \times 100\% \quad (5)$$

τ_{amp} is achieved by the function of:

$$\tau_{amp} = \sum_i \varphi_i^{amp} \tau_i \quad (6)$$

Synthesis of *R/S*-COOCz: Carbazole (3 g, 0.018 mol) and sodium hydride (NaH, 0.518 g, 0.022 mol) were placed into a 250 mL double-necked flask under an argon atmosphere, then 25 mL of tetrahydrofuran (THF) was added under an ice water bath and the mixture was stirred for 1 h at room temperature. Subsequently, methyl *S/R* -2-chloropropionate (2.4 mL, 0.022 mol) was rapidly injected into the reaction flask and stirred for 12 h at room temperature. After the reaction, 20 mL deionized water was added to the resulting solution and the mixture was extracted with dichloromethane (DCM) for three times (200 mL). The organic layer was collected and dried with anhydrous sodium sulfate. After filtration and solvent evaporation, the given residue was purified through silica gel column chromatography using DCM/ petroleum ether (PE) (V/V: 1/1) as eluent, and white solid of *R/S*-COOCz was obtained.

R-COOCz: ^{1}H NMR (400 MHz, $CDCl_3$) δ 8.13 (d, *J* = 7.5 Hz, 2H), 7.47 (t, *J* = 7.7 Hz, 2H), 7.39 (d, *J* = 8.0 Hz, 2H), 7.27 (t, *J* = 7.4 Hz, 2H), 5.44 (q, *J* = 7.3 Hz, 1H), 3.70 (s, 3H), 1.85 (d, *J* = 7.3 Hz, 3H). ^{13}C NMR (101 MHz, $CDCl_3$) δ = 171.78, 139.63, 125.96, 123.57, 120.61, 119.58, 109.29, 52.81, 52.16, 15.53. Yield: 3.06 g (66.7%).

S-COOCz: ^{1}H NMR (400 MHz, $CDCl_3$) δ 8.12 (d, *J* = 7.8 Hz, 2H), 7.47 (t, *J* = 7.6 Hz, 2H), 7.38 (d, *J* = 8.2 Hz, 2H), 7.27 (t, *J* = 7.3 Hz, 2H), 5.44 (q, *J* = 7.2 Hz, 1H), 3.70 (s, 3H), 1.85 (d, *J* = 7.2 Hz, 3H). ^{13}C NMR (101 MHz, $CDCl_3$) δ = 171.80, 139.66, 125.97, 123.59, 120.61, 109.30, 52.80, 52.18, 15.54. Yield: 2.8 g (61.1%).

Synthesis of *R*/*S*-COOHCz: *R*/*S*-COOCz (1 g, 0.004 mol) was placed into a 250 mL double-necked flask, and the 20 mL THF and 10 mL methanol was added and the mixture was stirred at room temperature for 10 min. After the solid was completely dissolved, 20 mL of sodium hydroxide (NaOH) solution (0.074 g/mL) was added by syringe and the mixture was stirred at 55 °C for 4 h. After the mixture was cooled down, the organic solvent was evaporated. Subsequently, acidification with concentrated hydrochloric acid (12 mol/L) precipitated the yellow solid, which was filtered with a glass filter funnel, washed with deionized water and dried in a vacuum oven overnight. The gray solid was obtained and used directly without further purification.

R-COOHCz: ^{1}H NMR (400 MHz, DMSO) δ 13.07 (s, 1H), 8.15 (d, *J* = 7.7 Hz, 2H), 7.52 (d, *J* = 8.2 Hz, 2H), 7.42 (t, *J* = 7.6 Hz, 2H), 7.20 (t, *J* = 7.4 Hz, 2H), 5.76 (q, *J* = 6.9 Hz, 1H), 1.67 (d, *J* = 7.1 Hz, 3H). ^{13}C NMR (101 MHz, DMSO) δ = 172.66, 139.64, 126.07, 122.79, 120.64, 119.40, 110.08, 51.70, 15.62. Yield: 0.738 g (77.2%).

S-COOHCz: ^{1}H NMR (400 MHz, DMSO) δ 13.12 (s, 1H), 8.15 (d, *J* = 7.7 Hz, 2H), 7.52 (d, *J* = 8.2 Hz, 2H), 7.42 (t, *J* = 7.6 Hz, 2H), 7.20 (t, *J* = 7.4 Hz, 2H), 5.76 (q, *J* = 6.8 Hz, 1H), 1.67 (d, *J* = 7.1 Hz, 3H). ^{13}C NMR (101 MHz, DMSO) δ = 172.69, 139.66, 126.09, 122.81, 120.66, 119.43, 110.10, 51.73, 15.65. Yield: 0.655 g (68.5%).

Synthesis of *R/S*-VCOOCz: *R*/*S*-COOHCz (0.36 g, 0.0015 mol), 1-ethyl-(3-dimethylaminopropyl) carbonyls diimide hydrochloride (EDCI) (0.144 g, 0.00075 mol) and 4-dimethylaminopyridine (0.0915 g, 0.00075 mol) were placed into a 250 mL double-necked flask and 20 mL of dichloromethane was added under an ice water bath and the mixture was stirred for 20 min. After

the solid was completely dissolved, acrylic acid-2-hydroxyethyl ester (0.209 g, 0.0018 mol) was added by syringe and stirred for 12 h at room temperature. After the reaction, the mixture was extracted with DCM for three times. The organic layer was collected and dried with anhydrous sodium sulfate. After filtration and solvent evaporation, the given residue was purified through silica gel column chromatography using ethyl acetate (EA)/PE (V/V: 1/3) as eluent, and yellow oil was obtained.

R-VCOOCz: ^{1}H NMR (400 MHz, $CDCl_3$) δ 8.08 (d, *J* = 7.7 Hz, 2H), 7.42 (t, *J* = 7.6 Hz, 2H), 7.36 (d, *J* = 8.2 Hz, 2H), 7.24 (t, *J* = 7.3 Hz, 2H), 6.20 (d, *J* = 17.3 Hz, 1H), 5.90 (dd, *J* = 17.2, 10.4 Hz, 1H), 5.74 (d, *J* = 10.4 Hz, 1H), 5.44 (q, *J* = 7.2 Hz, 1H), 4.34 (d, *J* = 4.8 Hz, 2H), 4.25 (dt, *J* = 8.8, 4.4 Hz, 1H), 4.16 (dd, *J* = 12.8, 7.4 Hz, 1H), 1.84 (d, *J* = 7.3 Hz, 3H). ^{13}C NMR (101 MHz, DMSO) δ = 170.75, 165.24, 139.29, 125.81, 122.69, 120.42, 119.27, 109.80, 63.06, 62.00, 51.48, 15.36. Yield: 0.233 g (46%).

S-VCOOCz: ^{1}H NMR (400 MHz, $CDCl_3$) δ 8.10 (d, *J* = 7.7 Hz, 2H), 7.44 (t, *J* = 7.6 Hz, 2H), 7.37 (d, *J* = 8.2 Hz, 2H), 7.25 (t, *J* = 7.3 Hz, 2H), 6.21 (d, *J* = 17.3 Hz, 1H), 5.91 (dd, *J* = 17.2, 10.4 Hz, 1H), 5.76 (d, *J* = 10.4 Hz, 1H), 5.45 (q, *J* = 7.1 Hz, 1H), 4.36 (d, *J* = 4.6 Hz, 2H), 4.26 (dt, *J* = 8.5, 4.3 Hz, 1H), 4.18 (dd, *J* = 12.8, 7.4 Hz, 1H), 1.85 (d, *J* = 7.2 Hz, 3H). ^{13}C NMR (101 MHz, DMSO) δ = 171.19, 165.81, 139.63, 126.25, 123.05, 120.77, 119.75, 110.08, 63.45, 62.37, 51.92, 15.66. Yield: 0.165 g (32.7%).

General procedure of radical polymerization: In an argon atmosphere, 0.01 equivalent (eq) of 2,2'-azobis(2-methylpropionitrile) (AIBN) and 1.0 eq of vinyl derivative were dissolved in 25 mL freshly distilled THF under ice water. After the solid was completely dissolved, the mixture was gradually heated to 55°C and stirred for 16 h. After the reaction, the mixture was added dropwise to 200 mL methanol to precipitate polymeric materials, then the crude product was filtered, followed by washing with PE and DCM, acetone in sequence. Then the solid was dissolved in deionized water and dialyzed by a dialysis tube (molecular weight cut-off = 1000) for 72 h.

***S*-PAMCOOCz$_1$**: Following the general procedure of radical polymerization using *S*-VCOOCz (0.337 g, 1.0 mmol, 1.00 eq), acrylamide (AM, 3.55 g, 50.0 mmol, 50 eq), and appropriate amount

of AIBN (0.0836 g, 0.51 mmol, 0.51 eq) in 25 mL freshly distilled THF to afford 3.31 g white powder polymer with a yield of 85.2%. Mn = 16817 Da; Mw = 25661 Da; PDI = 1.53.

***S*-PAMCOOCz$_2$**: Following the general procedure of radical polymerization using *S*-VCOOCz (0.169 g, 0.5 mmol, 1.00 eq), acrylamide (3.55 g, 50.0 mmol, 100 eq), and appropriate amount of AIBN (0.0828 g, 0.505 mmol, 1.01 eq) in 25 mL freshly distilled THF to afford 3.51 g white powder polymer with a yield of 94.5%. Mn = 23603 Da; Mw = 38866 Da; PDI = 1.65.

***R*-PAMCOOCz$_2$**: Following the general procedure of radical polymerization using *R*-VCOOCz (0.169 g, 0.5 mmol, 1.00 eq), acrylamide (3.55 g, 50.0 mmol, 100 eq), and appropriate amount of AIBN (0.0828 g, 0.505 mmol, 1.01 eq) in 25 mL freshly distilled THF to afford 3.56 g white powder polymer with a yield of 95.8%. Mn = 22006 Da; Mw = 38117 Da; PDI = 1.73.

***S*-PAMCOOCz$_3$**: Following the general procedure of radical polymerization using *S*-VCOOCz (0.0843 g, 0.25 mmol, 1.00 eq), acrylamide (3.55 g, 50.0 mmol, 200 eq), and appropriate amount of AIBN (0.0824 g, 0.5025 mmol, 2.01 eq) in 25 mL freshly distilled THF to afford 3.51 g white powder polymer with a yield of 96.6%. Mn = 25102 Da; Mw = 41739 Da; PDI = 1.66.

***S*-PAMCOOCz$_4$**: Following the general procedure of radical polymerization using *S*-VCOOCz (0.0421 g, 0.125 mmol, 1.00 eq), acrylamide (3.55 g, 50.0 mmol, 400 eq), and appropriate amount of AIBN (0.0822 g, 0.5013 mmol, 4.01 eq) in 25 mL freshly distilled THF to afford 3.50 g white powder polymer with a yield of 97.4%. Mn = 29095 Da; Mw = 46679 Da; PDI = 1.60.

Preparation of full-color polymer films: 0.5 g of polymer powder and a certain amount of organic fluorescent dyes were dissolved in deionized water (10 mL) followed by the vigorous sonication for 10 min under ambient conditions; then the solution was poured into a flask and stirred at 60°C for 1 h to obtain a completely transparent polymer solution; finally the mixed solution was placed in a petri dish and dried in an oven at 70°C overnight to fabricate transparent polymer films for subsequent photophysical and morphological characterizations.

Supplementary Figures and Tables

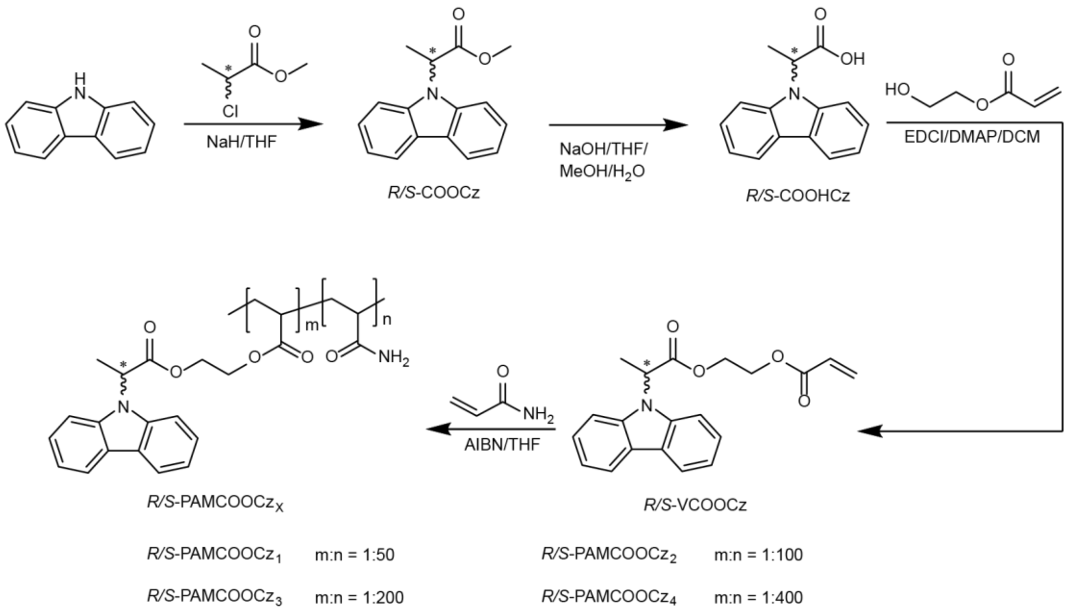

Supplementary Figure 1. Synthetic route of blue afterglow polymers *R*/S-PAMCOOCz$_X$ (X=1~4).

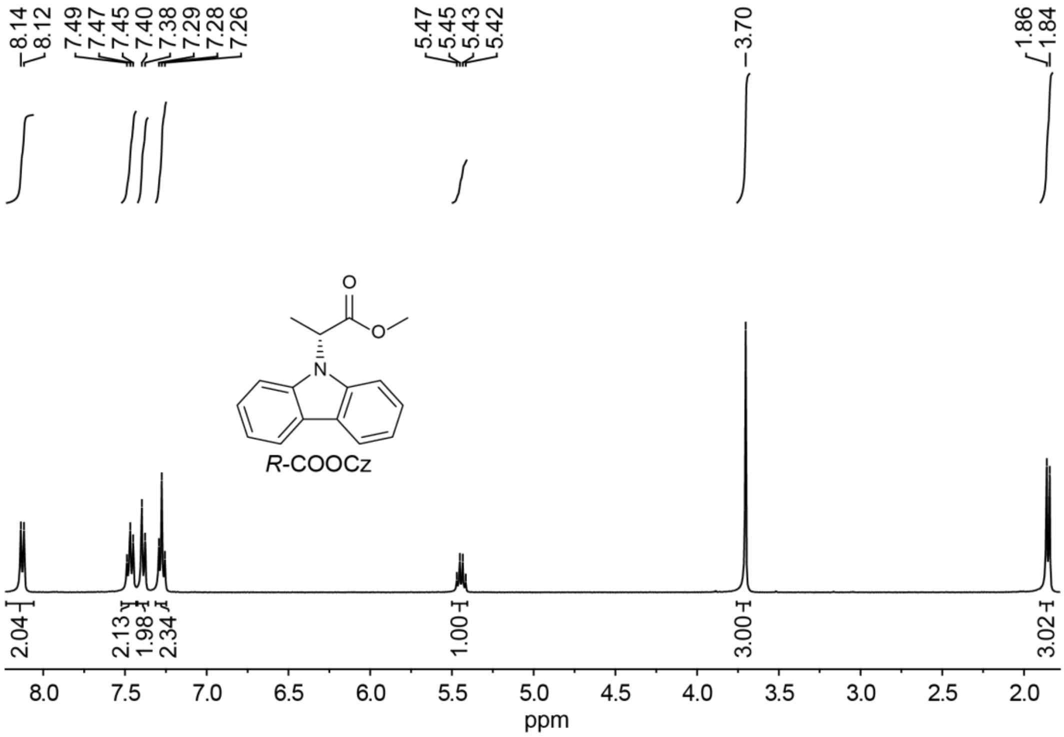

Supplementary Figure 2. ^{1}H NMR spectrum of *R*-COOCz in $CDCl_3$.

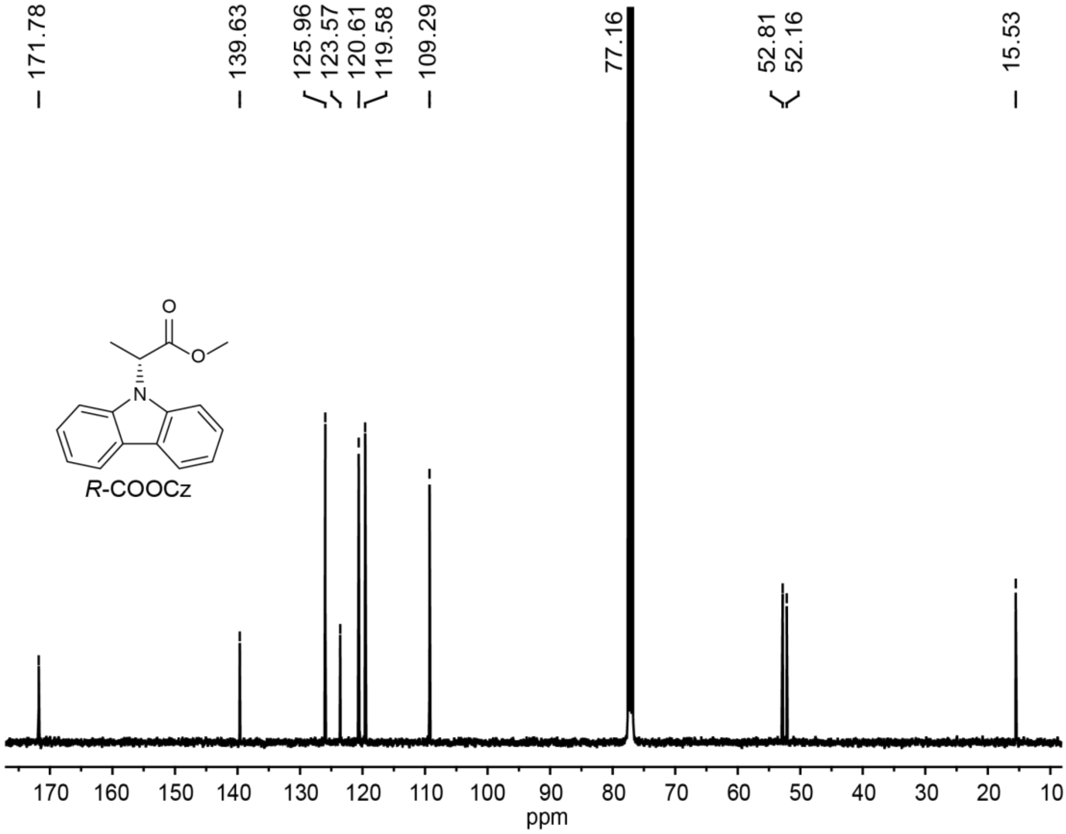

Supplementary Figure 3. ^{13}C NMR spectrum of *R*-COOCz in $CDCl_3$.

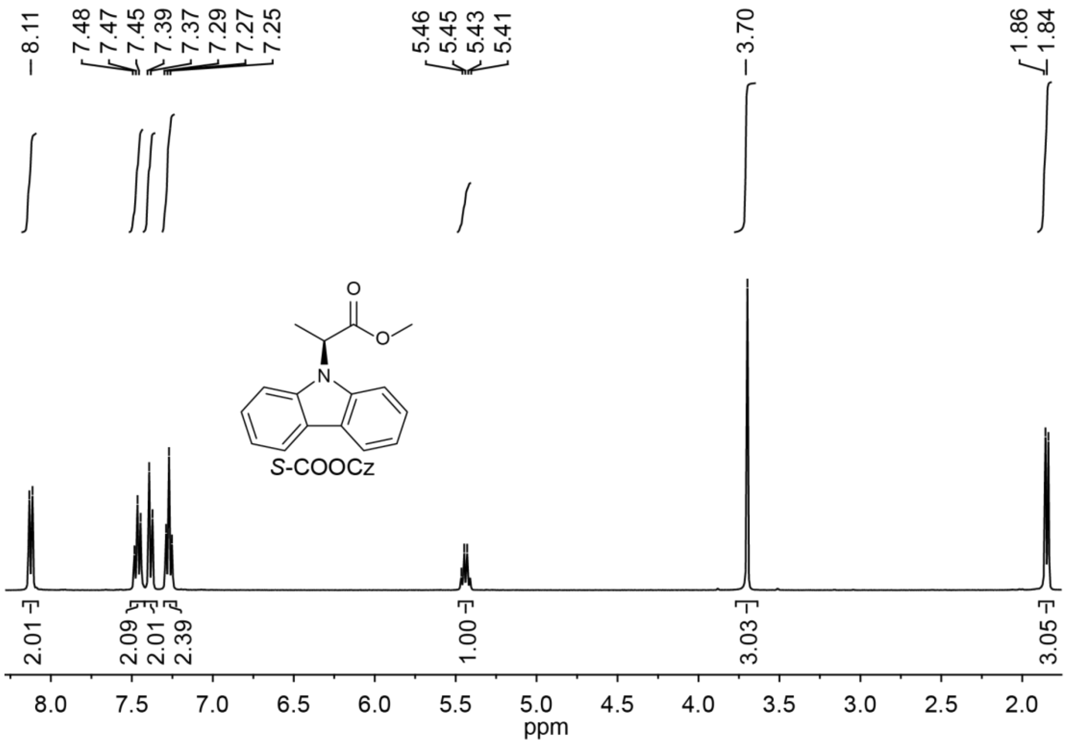

Supplementary Figure 4. ^{1}H NMR spectrum of *S*-COOCz in $CDCl_3$.

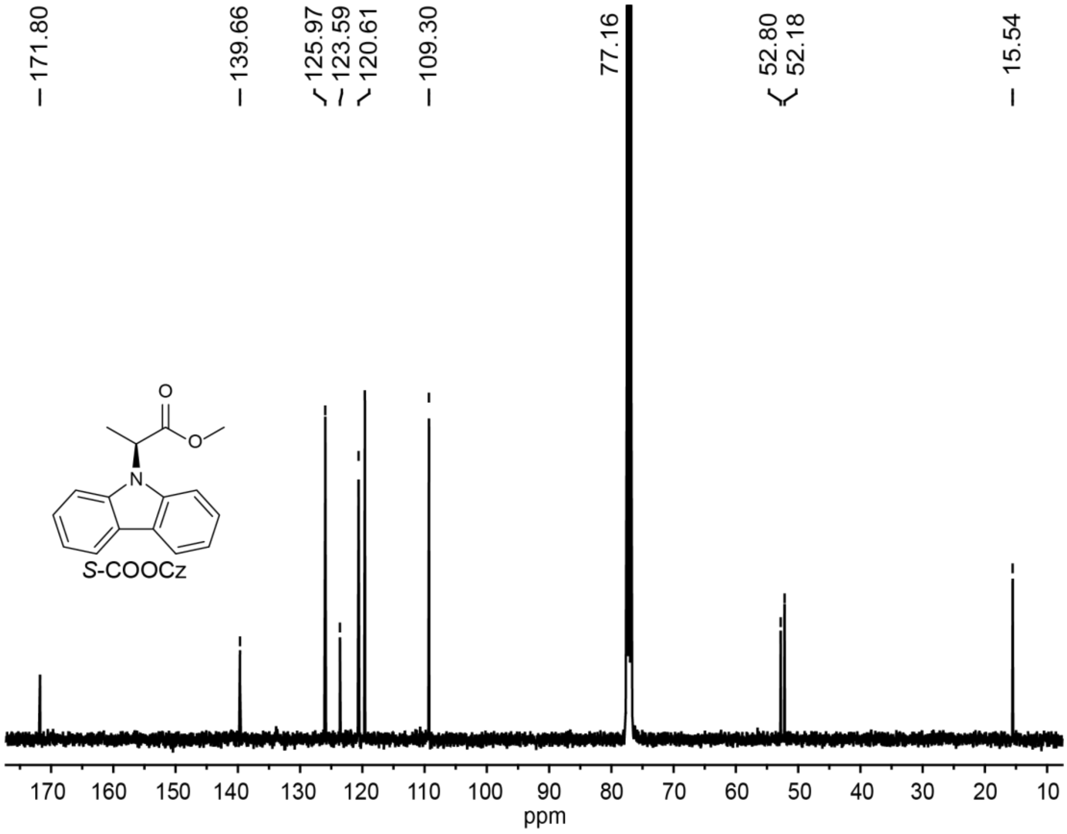

Supplementary Figure 5. ^{13}C NMR spectrum of *S*-COOCz in $CDCl_3$.

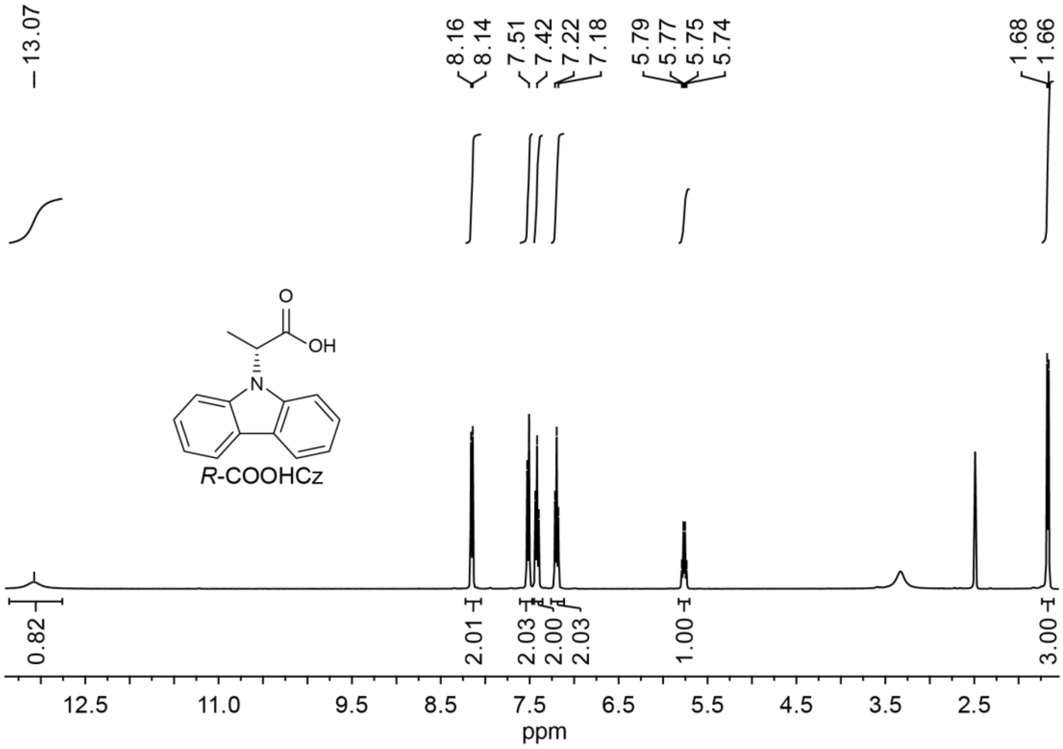

Supplementary Figure 6. ^{1}H NMR spectrum of *R*-COOHCz in DMSO-d_6.

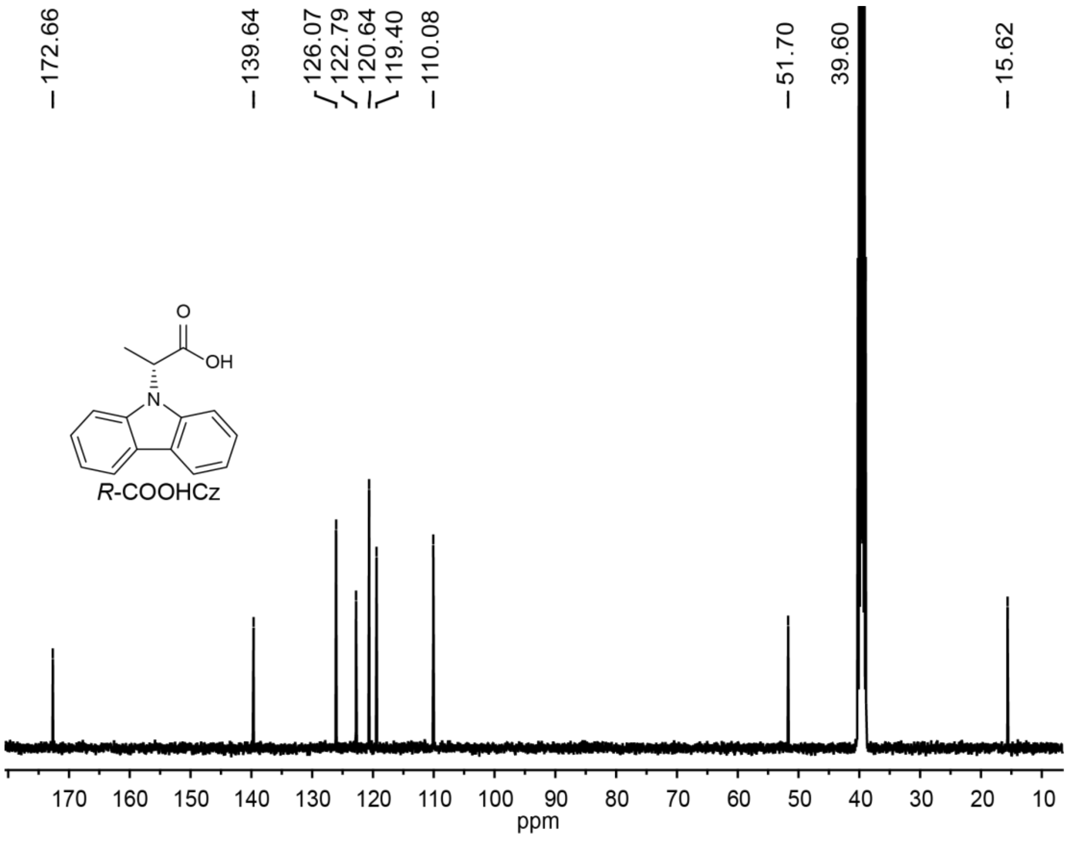

Supplementary Figure 7. ^{13}C NMR spectrum of *R*-COOHCz in DMSO-d_6.

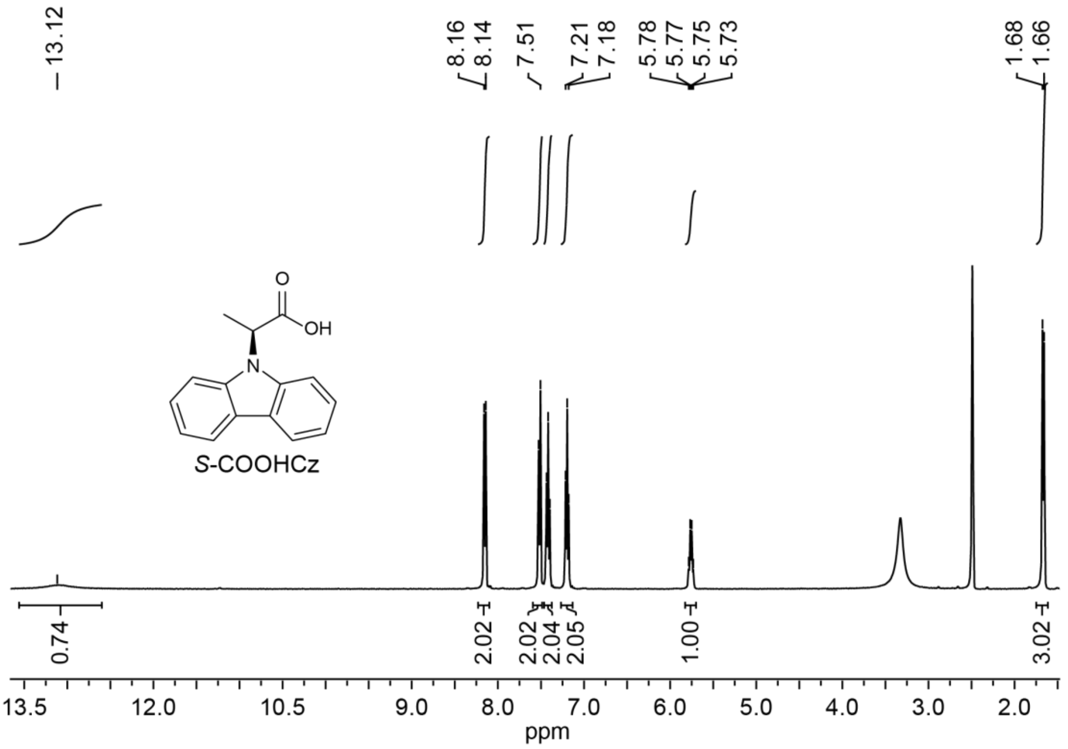

Supplementary Figure 8. ^{1}H NMR spectrum of *S*-COOHCz in DMSO-d_6.

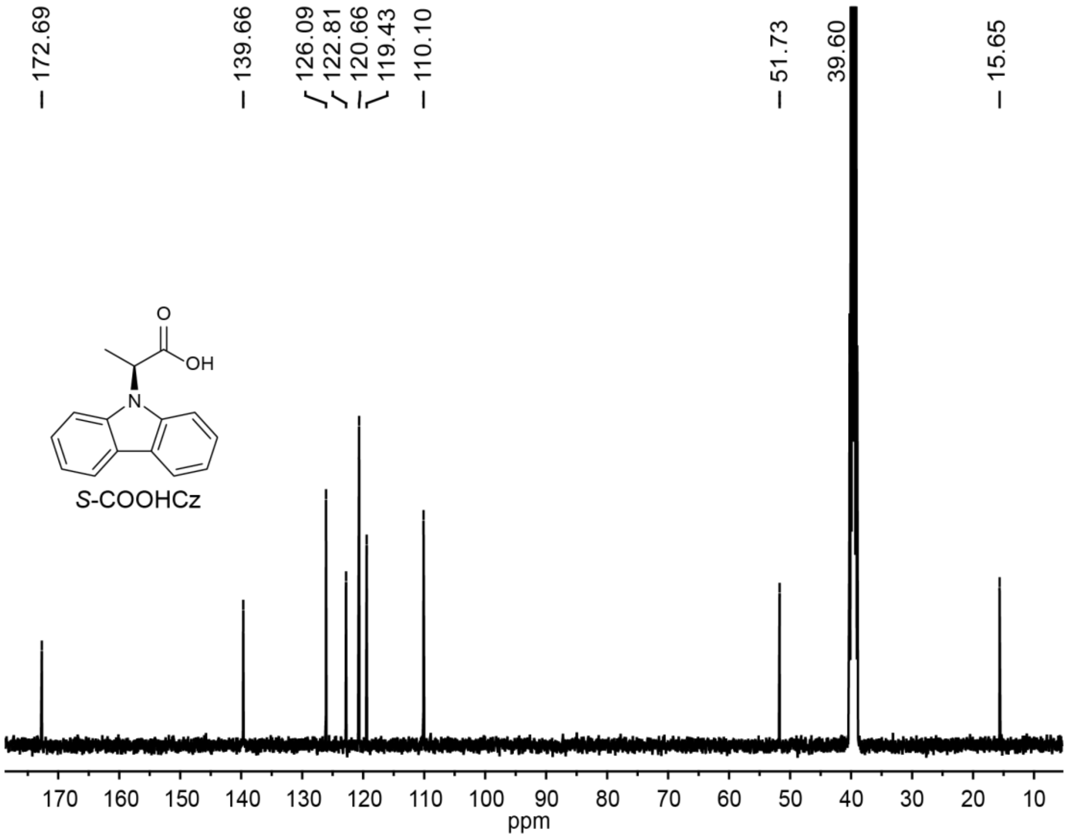

Supplementary Figure 9. ^{13}C NMR spectrum of *S*-COOHCz in DMSO-d_6.

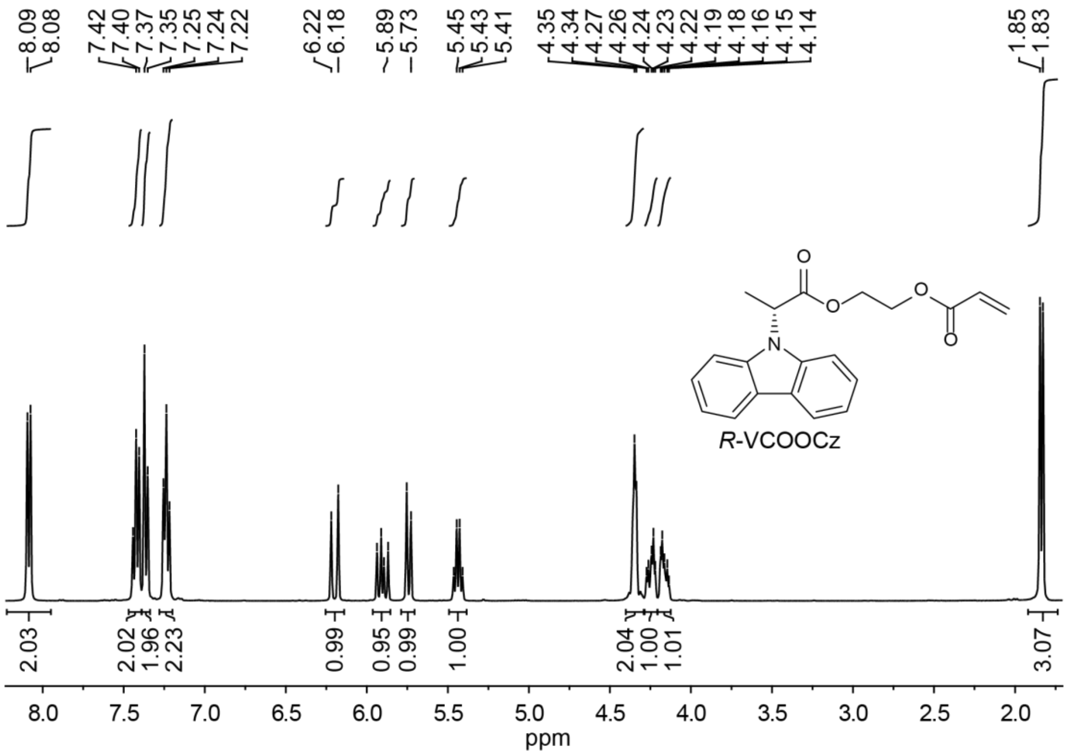

Supplementary Figure 10. ^{1}H NMR spectrum of *R*-VCOOCz in $CDCl_3$.

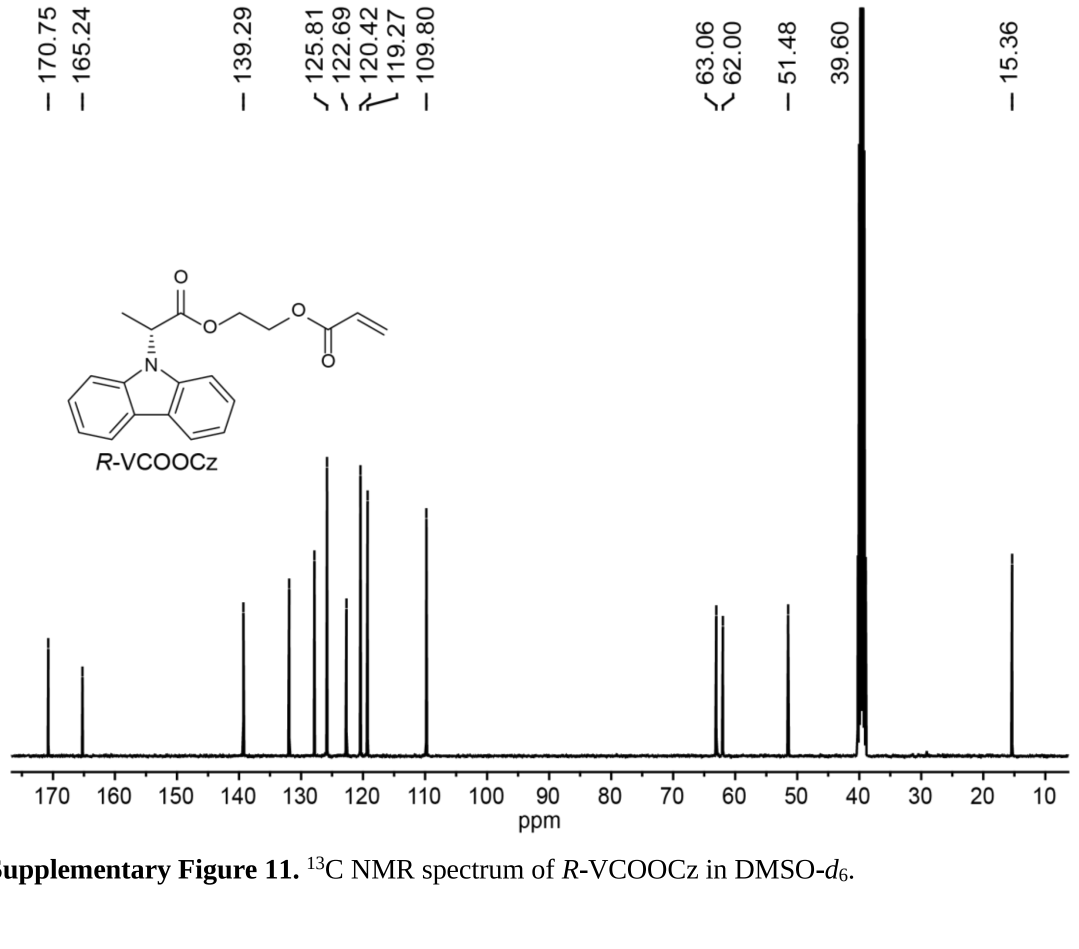

Supplementary Figure 11. ^{13}C NMR spectrum of *R*-VCOOCz in DMSO-d_6.

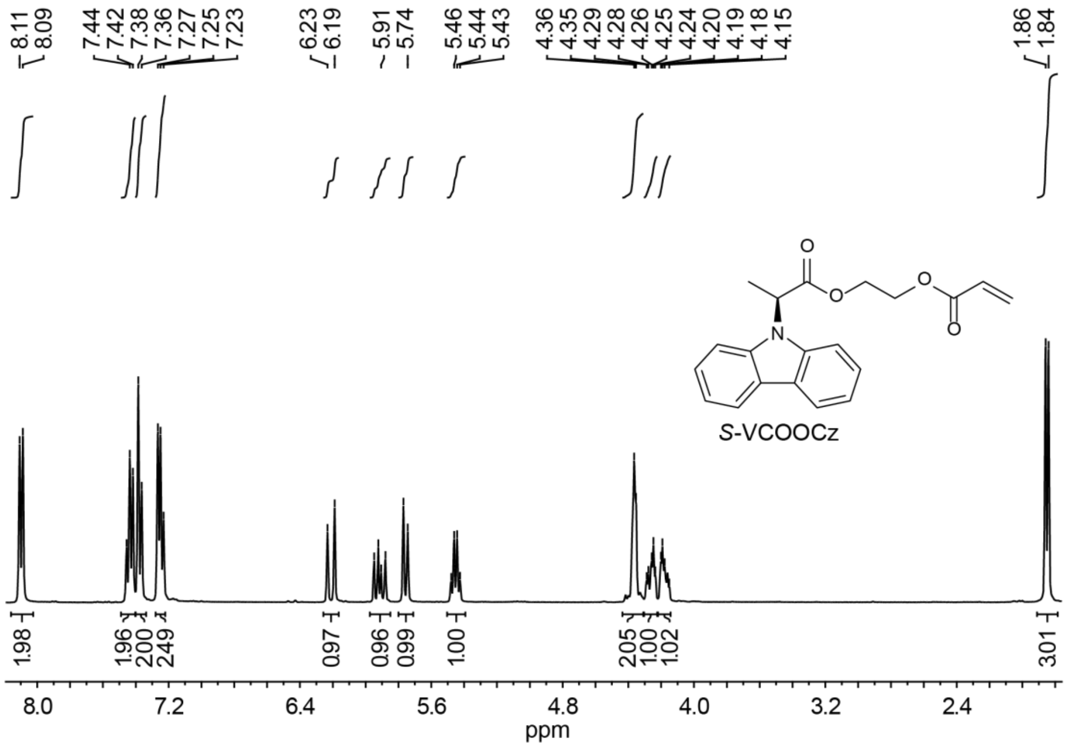

Supplementary Figure 12. ^{1}H NMR spectrum of *S*-VCOOCz in $CDCl_3$.

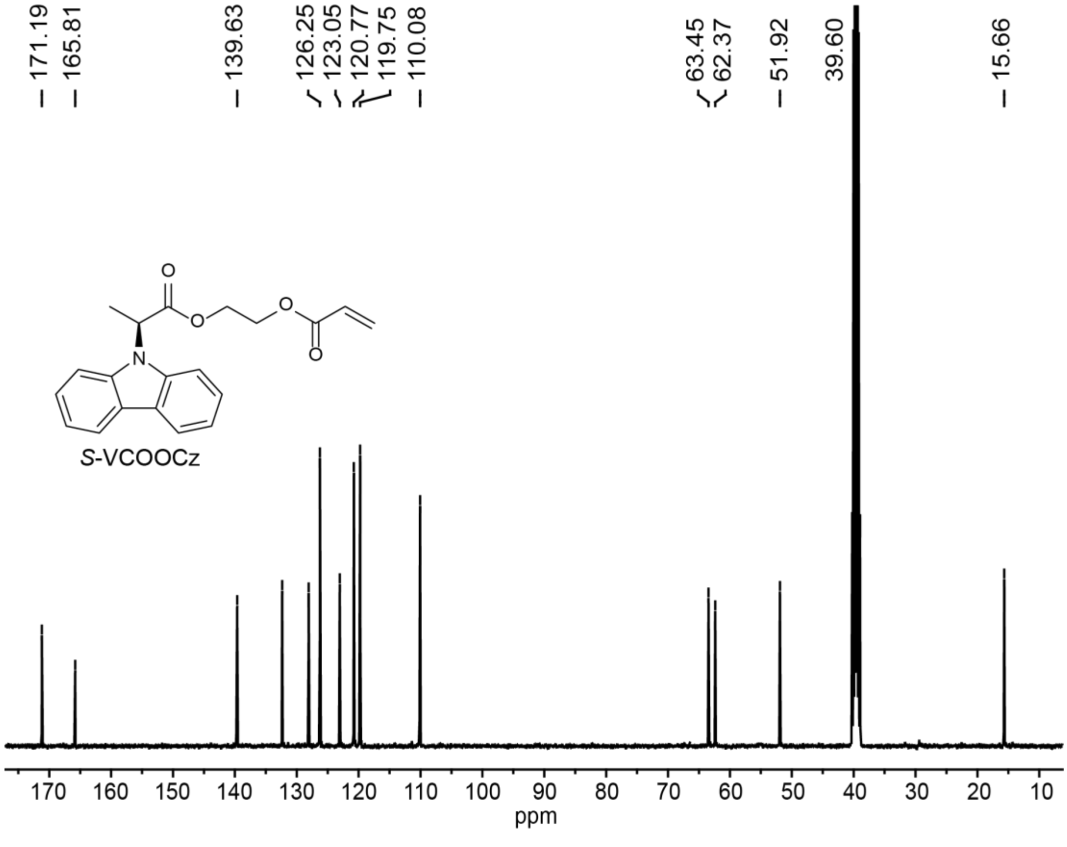

Supplementary Figure 13. ^{13}C NMR spectrum of *S*-VCOOCz in DMSO-d_6.

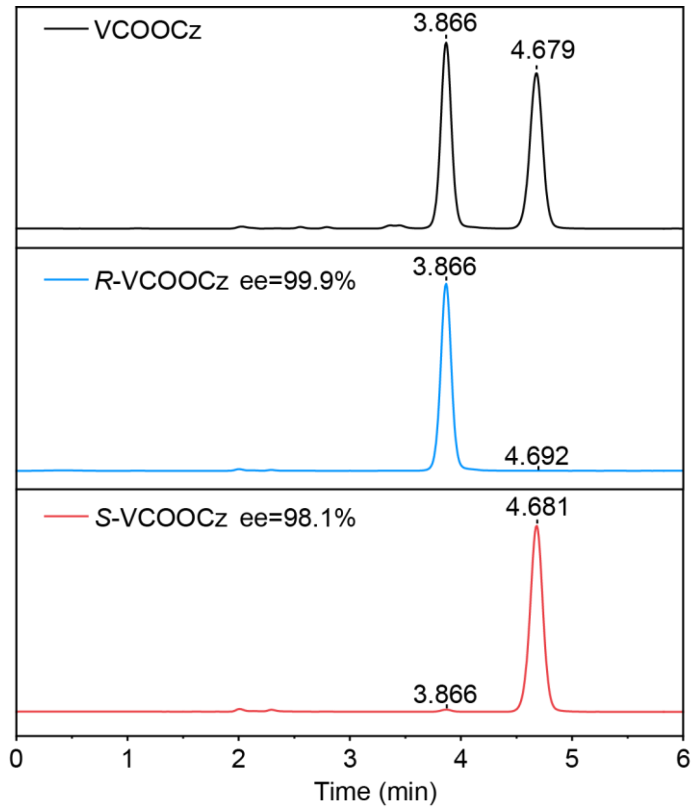

Supplementary Figure 14. HPLC profiles of racemic VCOOCz, *R*-VCOOCz and *S*-VCOOCz.

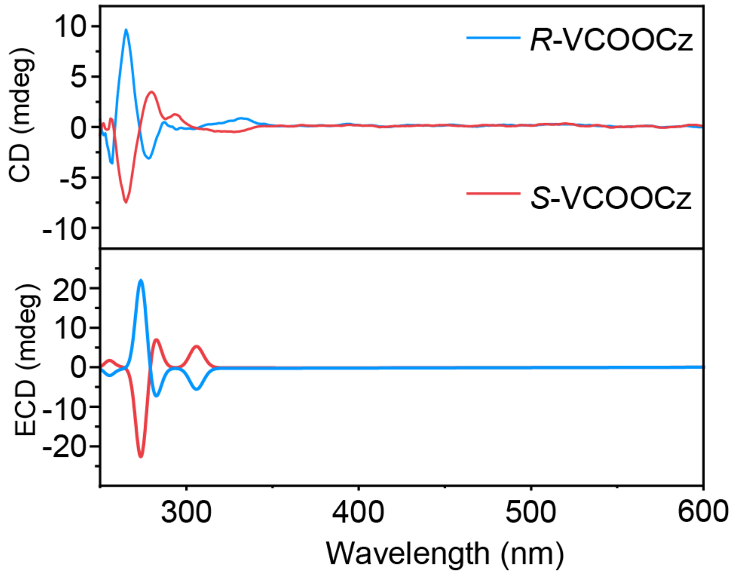

Supplementary Figure 15. Experimental (top panel) and calculated (bottom panel) CD spectra of *R/S*-VCOOCz.

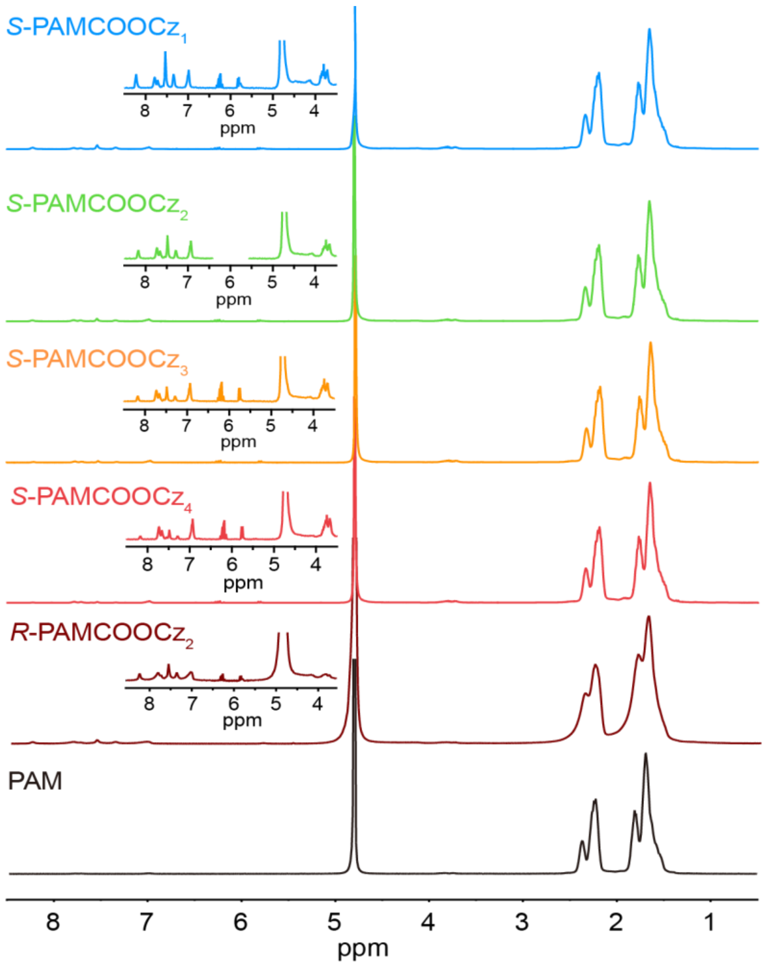

Supplementary Figure 16. ^{1}H NMR spectra of polyacrylamide (PAM), *R*-PAMCOOCz$_2$ and *S*-PAMCOOCz$_X$ (X=1~ 4) in D_2O.

Supplementary Table 1. Characterizations of polymers *R*-PAMCOOCz$_2$ and *S*-PAMCOOCz$_X$ (X=1~4) by GPC.

**Polymer**	M_n **(Daltons)**	M_w **(Daltons)**	**PDI**
S-PAMCOOCz$_1$	16817	25661	1.53
S-PAMCOOCz$_2$	23603	38866	1.65
R-PAMCOOCz$_2$	22006	38117	1.73
S-PAMCOOCz$_3$	25102	41739	1.66
S-PAMCOOCz$_4$	29095	46679	1.60

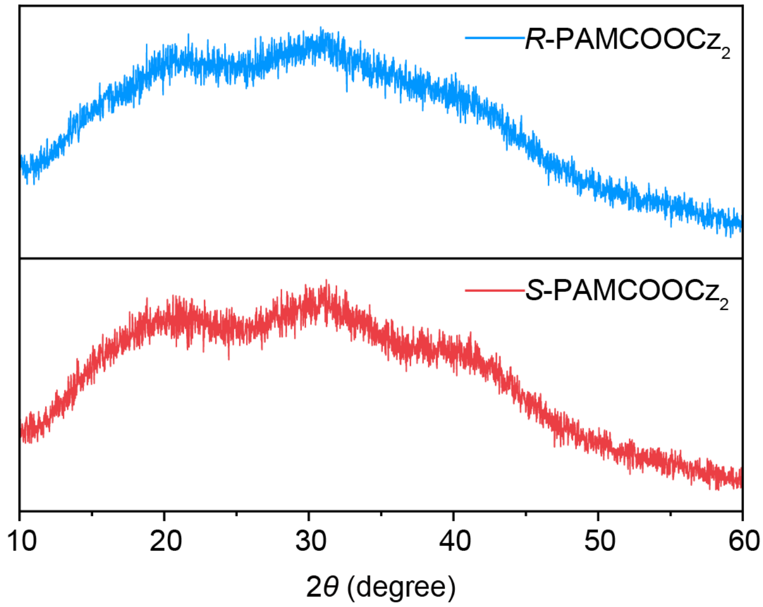

Supplementary Figure 17. Powder XRD spectra of *R*/*S*-PAMCOOCz$_2$ films.

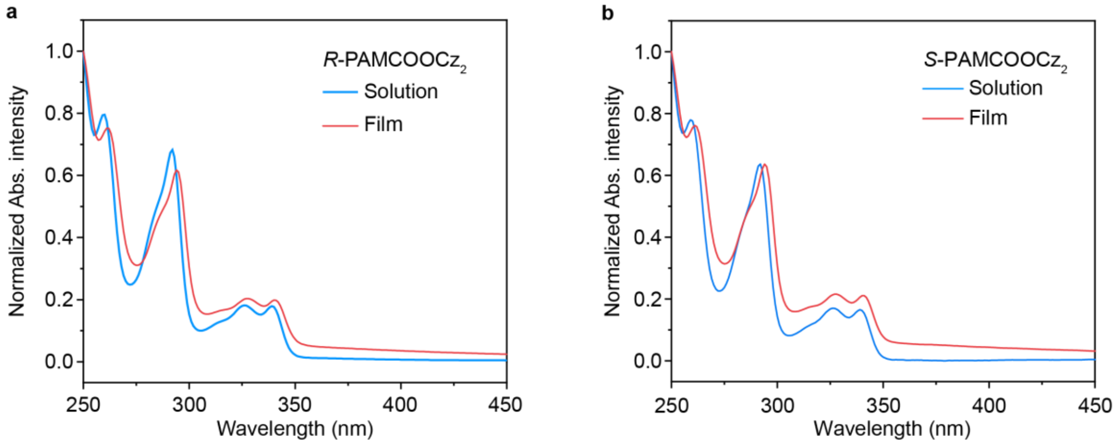

Supplementary Figure 18. Normalized absorption spectra of (a) *R*-PAMCOOCz$_2$ and (b) *S*-PAMCOOCz$_2$ in aqueous solution and film state.

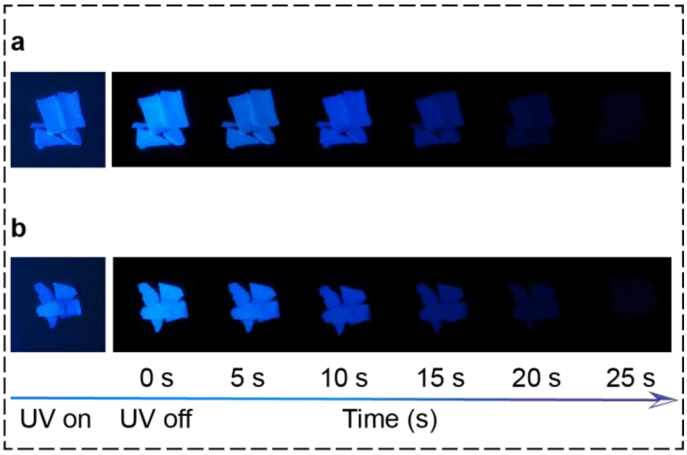

Supplementary Figure 19. Photographs of (a) *R*-PAMCOOCz$_2$ and (b) *S*-PAMCOOCz$_2$ films taken under and after the removal of the 254 nm excitation source.

Supplementary Table 2. Fluorescence and afterglow lifetimes of *R*/*S*-PAMCOOCz$_2$ films.

Sample	Fluorescence		Afterglow	
	λ (nm)	τ_{int} (ns)	λ (nm)	τ_{int} (s)
R-PAMCOOCz$_2$	364	12.9	414/442/470	3.0/3.1/3.1
S-PAMCOOCz$_2$	364	11.7	414/442/470	3.0/3.1/3.0

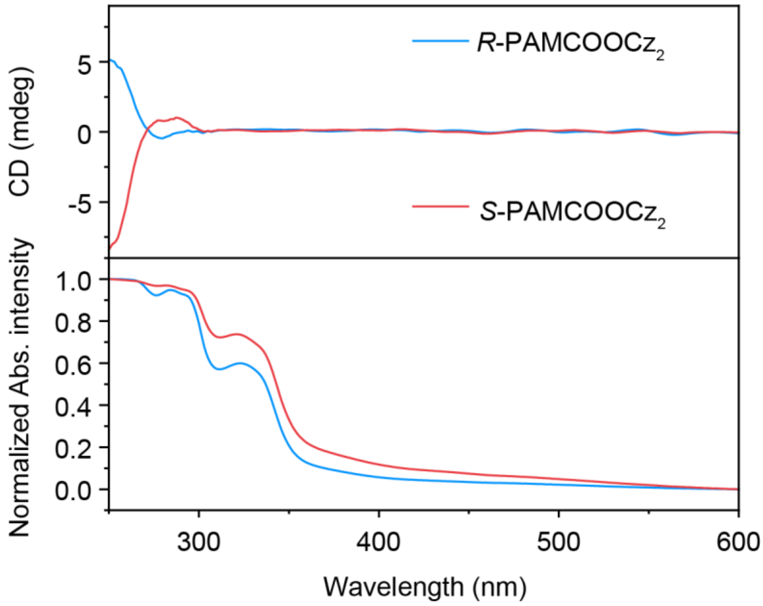

Supplementary Figure 20. Circular dichroism and absorption spectra of *R*/*S*-PAMCOOCz$_2$ films.

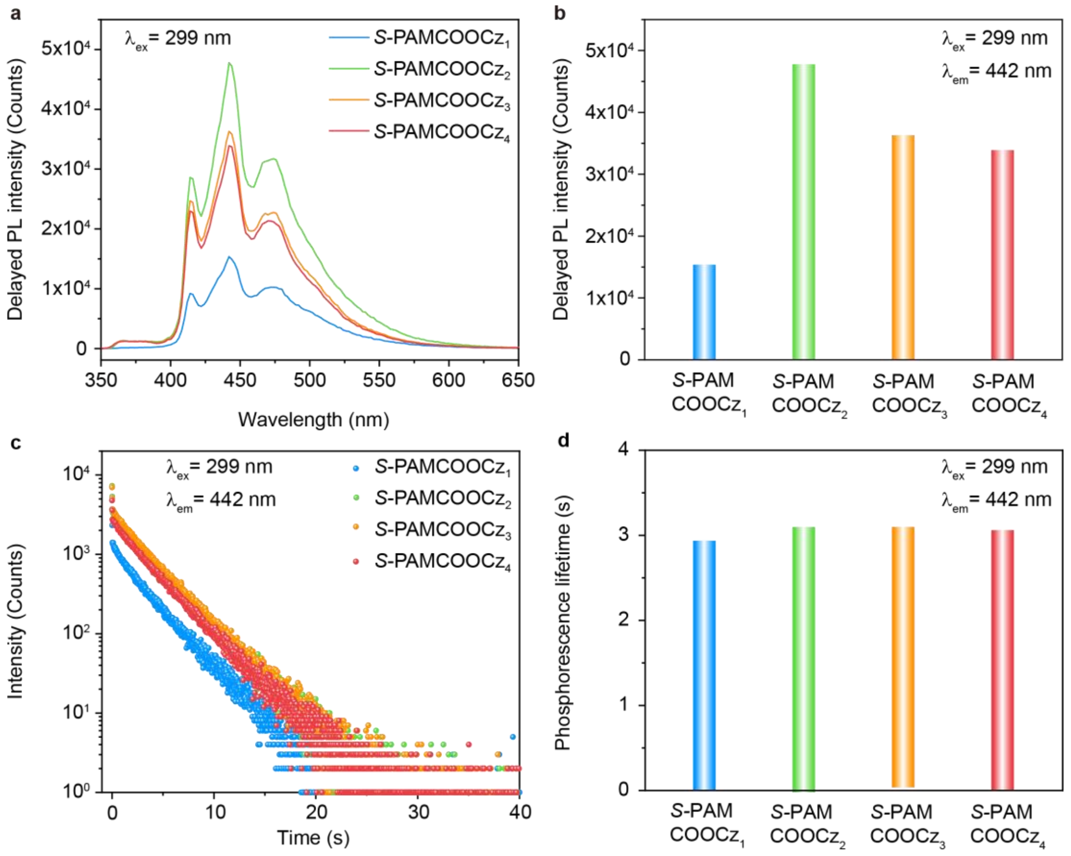

Supplementary Figure 21. (a, b) Delayed PL spectra (10 ms delay) (a) and corresponding afterglow intensities (b) of *S*-PAMCOOCz$_X$ （X=1~4） films at different molar feeding ratios between *S*-VCOOCz and AM. (c, d) Afterglow decay profiles (c) and corresponding lifetimes (d) of *S*-PAMCOOCz$_X$ films at different molar feeding ratios between *S*-VCOOCz and AM.

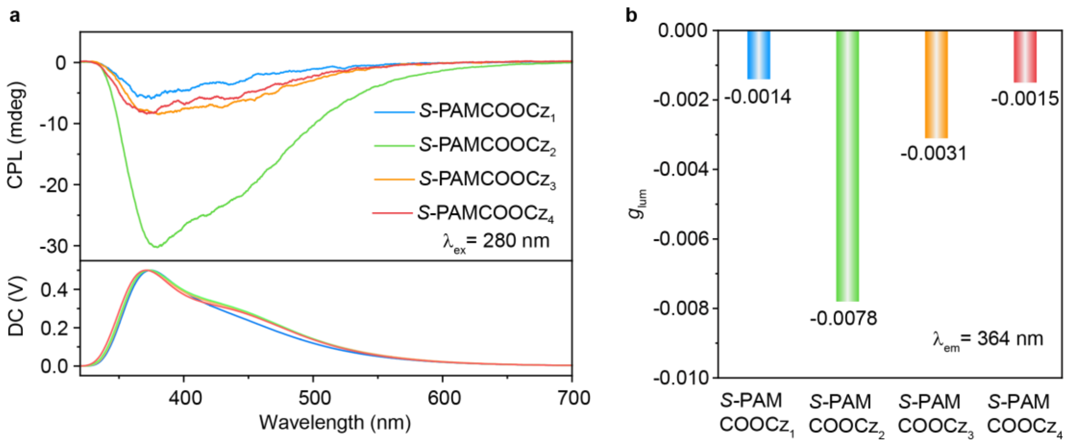

Supplementary Figure 22. (a) CPL spectra and (b) corresponding asymmetry factors (g_{lum}) of *S*-PAMCOOCz$_X$ (X=1~4) films at different molar feeding ratios between *S*-VCOOCz and AM excited by 280 nm UV light.

Supplementary Table 3. Photophysical properties of *S*-PAMCOOCz$_X$ (X=1~4) films excited by 299 nm UV light.

**Parameters**	*S*-PAMCOOCz$_1$	*S*-PAMCOOCz$_2$	*S*-PAMCOOCz$_3$	*S*-PAMCOOCz$_4$
Φ_{SSPL} (%)	22.08	24.70	26.04	26.43
Φ_F (%)	8.41	9.00	11.72	12.42
Φ_{OA} (%)	13.67	15.70	14.32	14.01
Φ_{ISC}^{min} (%)	13.67	15.70	14.32	14.01
Φ_{ISC}^{max} (%)	91.59	91.00	88.28	87.58
τ_{int}^{OA} (s)	2.94	3.12	3.06	3.05
$k_r{}^{OA}{}_{min}$ (s^{-1})	0.05	0.06	0.05	0.05
$k_r{}^{OA}{}_{max}$ (s^{-1})	0.34	0.32	0.33	0.33
$k_{nr}{}^{OA}{}_{min}$ (s^{-1})	0.00	0.00	0.00	0.00
$k_{nr}{}^{OA}{}_{max}$ (s^{-1})	0.29	0.27	0.27	0.28

Supplementary Table 4. Photoluminescence quantum yields (Φ_{SSPL}) of the *R*/*S*-PAMCOOCz_2 films without and with doping of different fluorescent guests.

**Samples**	**Φ_{SSPL} (%)**
R-PAMCOOCz_2	28.6
0.05 wt.% Fluc/*R*-PAMCOOCz_2	23.3
0.07 wt.% Fluc/*R*-PAMCOOCz_2	25.7
0.1 wt.% Fluc/*R*-PAMCOOCz_2	28.1
0.1 wt.% Rh123/*R*-PAMCOOCz_2	17.6
0.1 wt.% SR101/*R*-PAMCOOCz_2	37.3
S-PAMCOOCz_2	24.7
0.05 wt.% Fluc/*S*-PAMCOOCz_2	24.3
0.07 wt.% Fluc/*S*-PAMCOOCz_2	26.6
0.1 wt.% Fluc/*S*-PAMCOOCz_2	31.6
0.1 wt.% Rh123/*S*-PAMCOOCz_2	20.2
0.1 wt.% SR101/*S*-PAMCOOCz_2	20.9

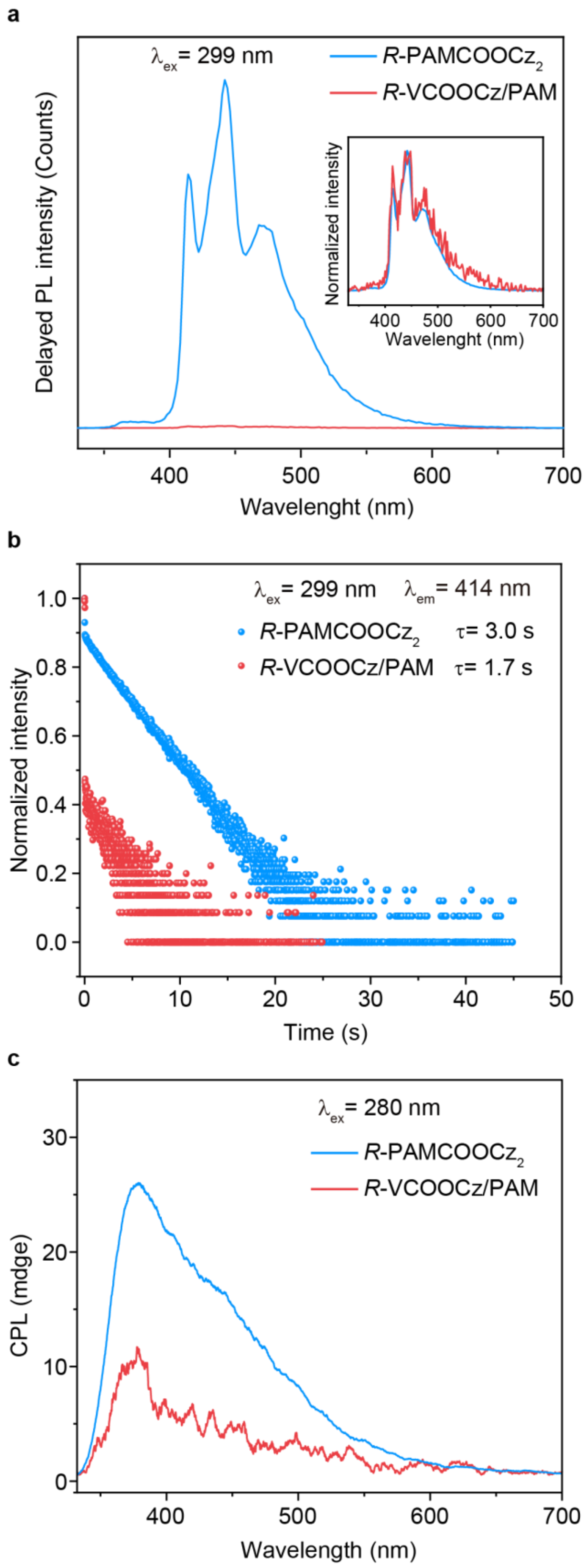

Supplementary Figure 23. (a) Delayed PL (10 ms delay), (b) afterglow lifetime and (c) CPL profiles of *R*-PAMCOOCz$_2$ and *R*-VCOOCz/PAM films. Insert shows the normalized delayed PL spectra.

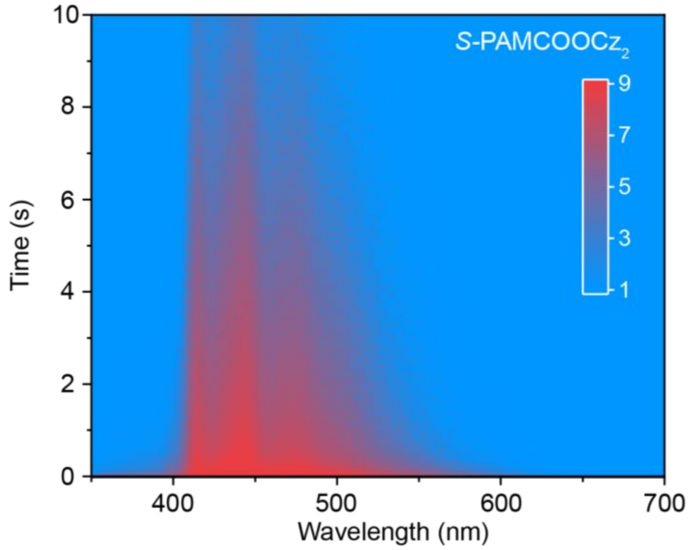

Supplementary Figure 24. TRES of *S*-PAMCOOCz_2 film excited by 299 nm UV light.

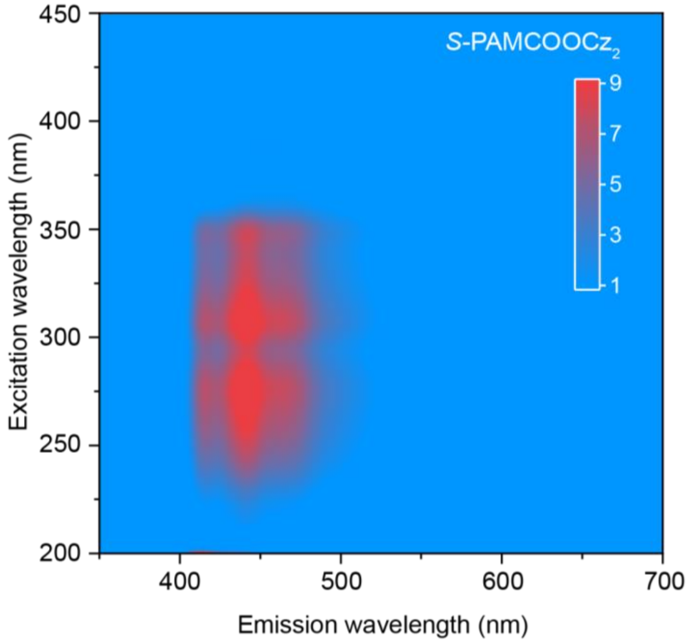

Supplementary Figure 25. Excitation-delayed PL emission spectra of *S*-PAMCOOCz_2 film.

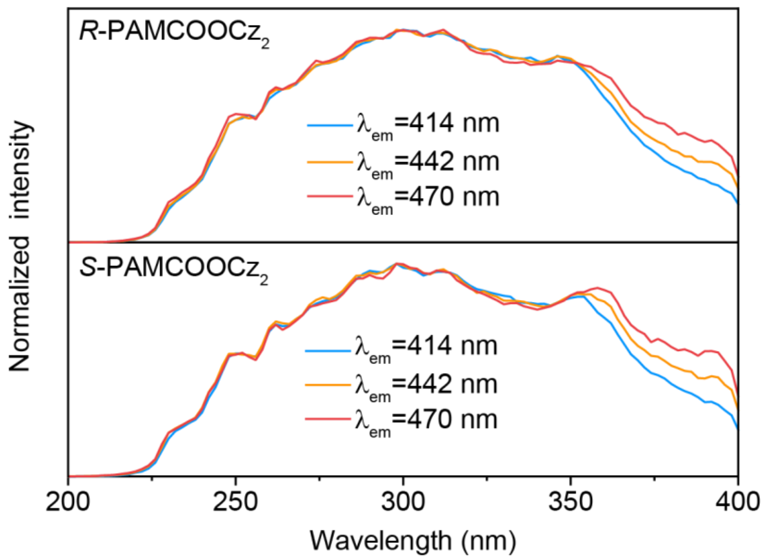

Supplementary Figure 26. Excitation spectra of *R*/*S*-PAMCOOCz$_2$ films by monitoring the afterglow emission peak at 414, 442 and 470 nm under ambient conditions respectively.

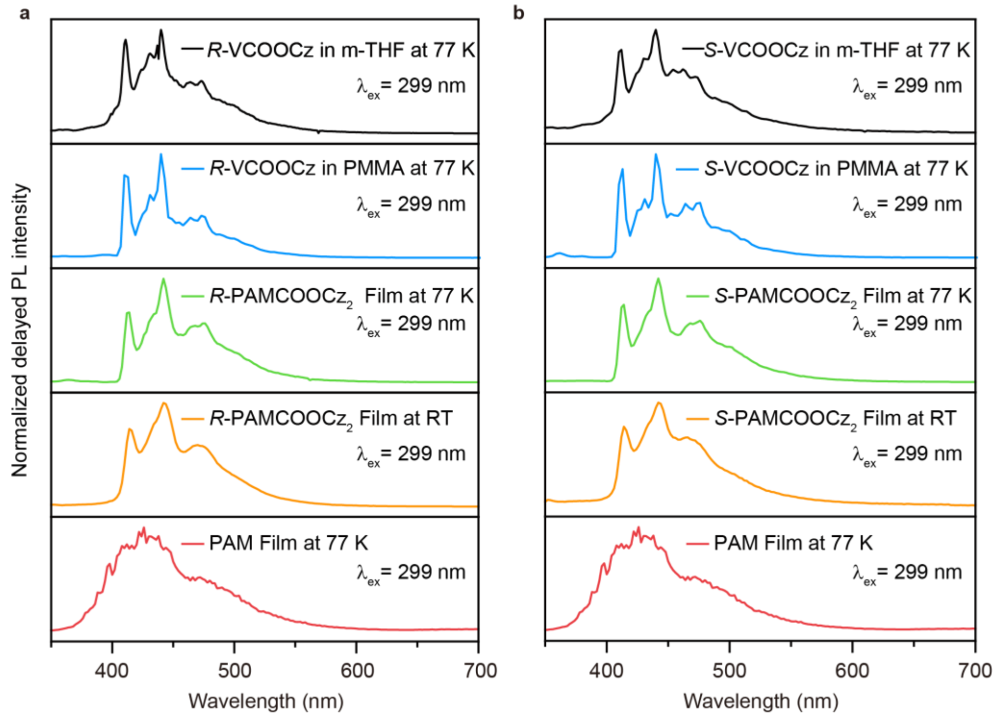

Supplementary Figure 27. (a) Delayed PL spectra (10 ms delay) of *R*-VCOOCz, *R*-PAMCOOCz$_2$ and PAM in varied states recorded at 77 K and room temperature. (b) Delayed PL spectra (10 ms delay) of *S*-VCOOCz, *S*-PAMCOOCz$_2$ and PAM in varied states recorded at 77 K and room temperature.

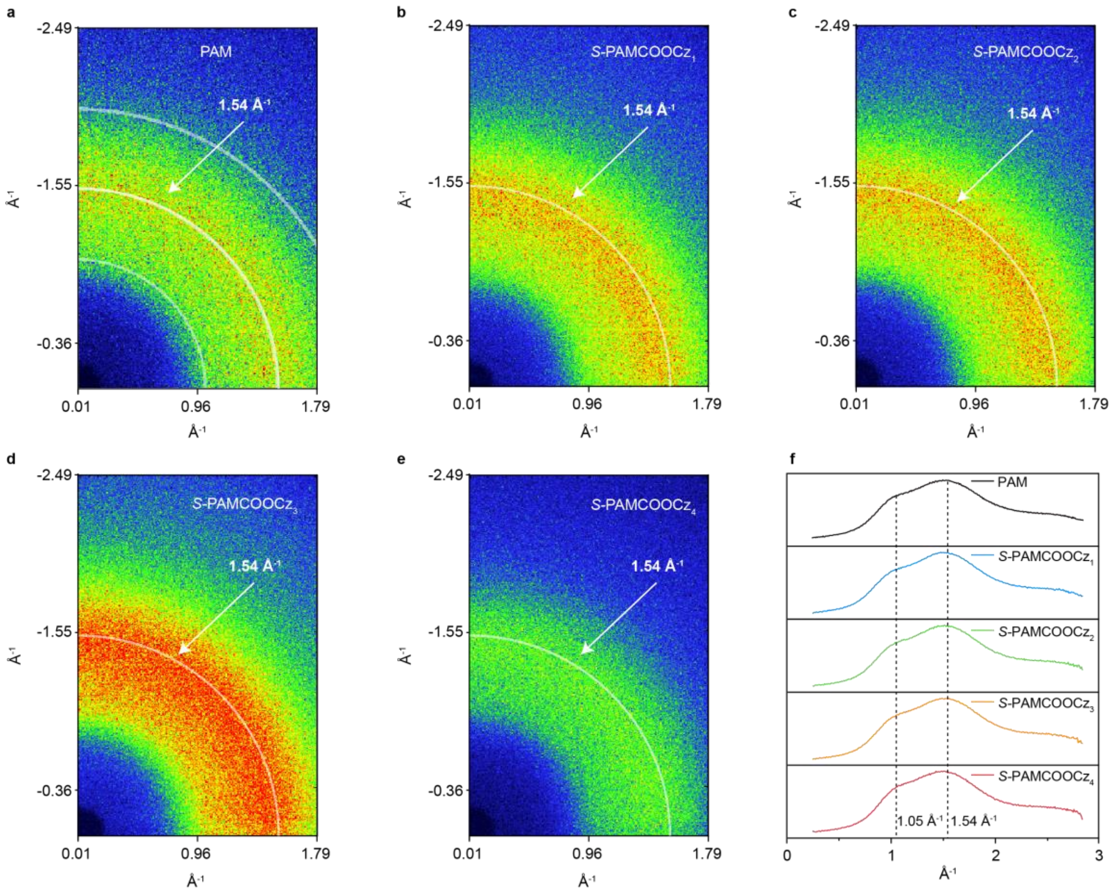

Supplementary Figure 28. 2D-WAXS patterns of (a) PAM, (b) *S*-PAMCOOCz$_1$, (c) *S*-PAMCOOCz$_2$, (d) *S*-PAMCOOCz$_3$ and (e) *S*-PAMCOOCz$_4$ films. (f) WAXS spectra of PAM, and *S*-PAMCOOCz$_X$ (X=1~4) polymer films.

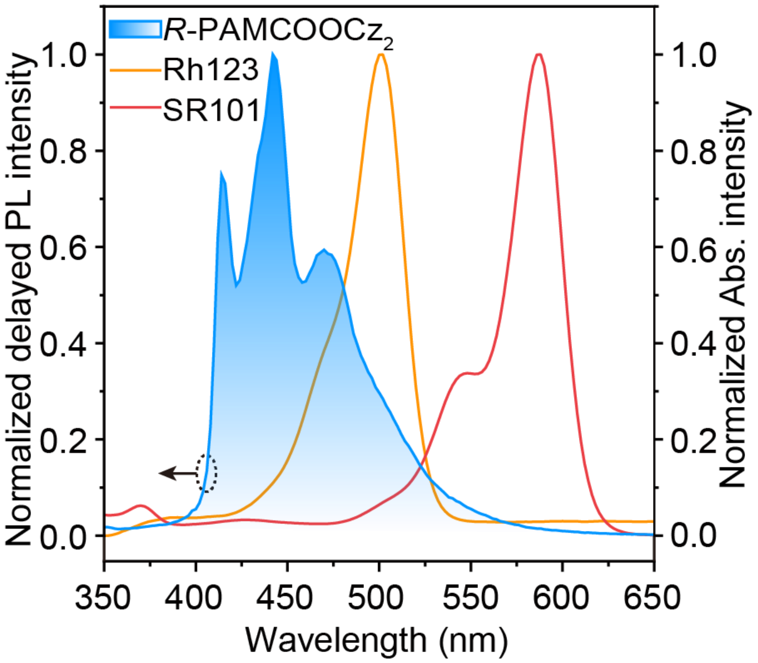

Supplementary Figure 29. Delayed PL spectra (10 ms delay) of *R*-PAMCOOCz$_2$ films (blue region, delayed time, 10 ms) and absorption spectra of aqueous solutions (10^{-5} mol/L) of Rh123 and SR101.

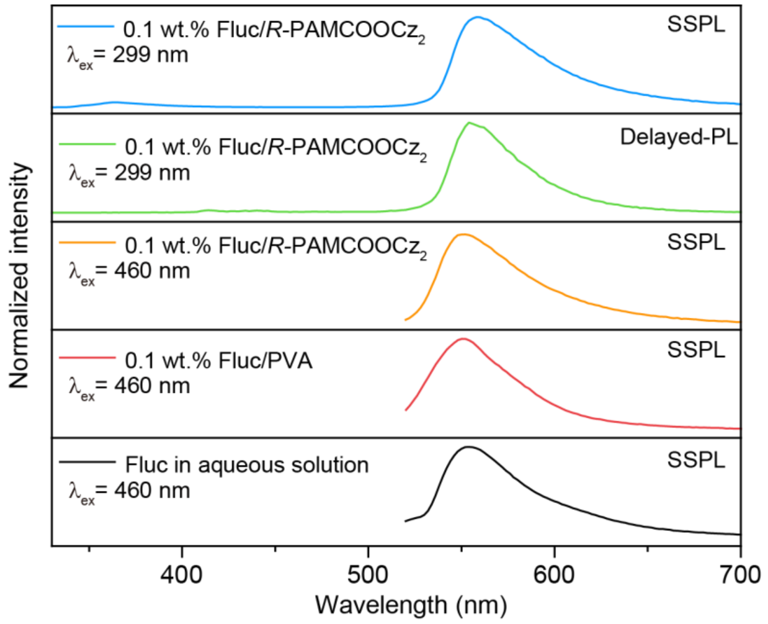

Supplementary Figure 30. SSPL and delayed PL spectra of Fluc in aqueous solution and doped films.

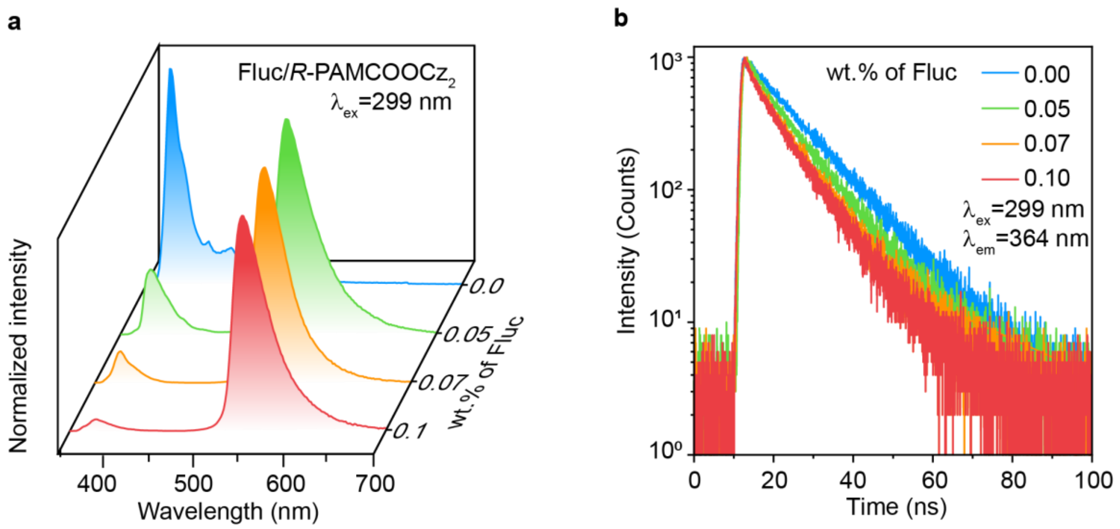

Supplementary Figure 31. (a) Fluc/*R*-PAMCOOCz$_2$ steady-state PL spectra at different concentrations. (b) Doped thin films at 364 nm at different mass concentrations fluorescence decay curves, λ_{ex} = 299 nm at room temperature.

Supplementary Table 5. Photophysical properties of *R*/*S*-$PAMCOOCz_2$ and Fluc-doped *R*/*S*-$PAMCOOCz_2$ films excited by 299 nm UV light source.

Samples	Wavelength (nm)	Fluorescence τ_1 (ns)	Fluorescence φ_1 (%)	Fluorescence τ_{amp} (ns)	Afterglow τ_1 (s)	Afterglow φ_1 (%)	Afterglow τ_2 (s)	Afterglow φ_2 (%)	Afterglow τ_{int} (s)	Afterglow τ_{amp} (s)
R-$PAMCOOCz_2$	364	12.9	100	12.9	-	-	-	-	-	-
	414	-	-	-	1.0	4.51	3.1	95.49	3.0	2.8
0.05 wt.% Fluc/*R*-$PAMCOOCz_2$	364	10.8	100	10.8	-	-	-	-	-	-
	414	-	-	-	0.2	9.58	2.7	90.42	2.4	1.3
	555	-	-	-	0.3	12.95	2.6	87.05	2.3	1.3
0.07 wt.% Fluc/*R*-$PAMCOOCz_2$	364	9.8	100	9.8	-	-	-	-	-	-
	414	-	-	-	0.3	13.14	2.4	86.86	2.1	1.2
	555	-	-	-	0.2	14.34	2.3	85.66	2.0	1.1
0.1 wt.% Fluc/*R*-$PAMCOOCz_2$	364	9.4	100	9.4	-	-	-	-	-	-
	414	-	-	-	0.2	11.81	2.3	88.19	2.0	1.1
	555	-	-	-	0.3	20.51	2.2	79.49	1.8	1.0
S-$PAMCOOCz_2$	364	12.1	100	12.1	-	-	-	-	-	-
	414	-	-	-	0.8	6.22	3.2	93.78	3.0	2.7
0.05 wt.% Fluc/*S*-$PAMCOOCz_2$	364	10.6	100	10.6	-	-	-	-	-	-
	414	-	-	-	0.2	8.00	2.8	92.00	2.6	1.5
	555	-	-	-	0.3	12.80	2.8	87.20	2.5	1.5
0.07 wt.% Fluc/*S*-$PAMCOOCz_2$	364	9.9	100	9.9	-	-	-	-	-	-
	414	-	-	-	0.2	9.74	2.6	90.26	2.4	1.3
	555	-	-	-	0.3	14.21	2.6	85.79	2.3	1.3
0.1 wt.% Fluc/*S*-$PAMCOOCz_2$	364	9.8	100	9.8	-	-	-	-	-	-
	414	-	-	-	0.2	10.01	2.5	89.99	2.3	1.2
	555	-	-	-	0.3	14.90	2.5	85.10	2.1	1.1

Supplementary Table 6. SACET efficiency of $PAMCOOCz_2$ films without or with doping of different fluorescence emitters at different weight concentrations.

**Samples**	**wt. (%)**	$\boldsymbol{\Phi_{P\text{-}SACET}}$ **(%)**	$\boldsymbol{\Phi_{F\text{-}SACET}}$ **(%)**
R-$PAMCOOCz_2$	0.00	--	--
Fluc/*R*-$PAMCOOCz_2$	0.05	53.6	16.3
Fluc/*R*-$PAMCOOCz_2$	0.07	57.1	24.0
Fluc/*R*-$PAMCOOCz_2$	0.10	64.3	27.1
Rh123/*R*-$PAMCOOCz_2$	0.10	36.4	
SR101/*R*-$PAMCOOCz_2$	0.10	17.1	
S-$PAMCOOCz_2$	0.00	--	
Fluc/*S*-$PAMCOOCz_2$	0.05	44.4	12.4
Fluc/*S*-$PAMCOOCz_2$	0.07	51.9	18.2
Fluc/*S*-$PAMCOOCz_2$	0.10	55.6	19.0
Rh123/*S*-$PAMCOOCz_2$	0.10	26.3	
SR101/*S*-$PAMCOOCz_2$	0.10	18.1	

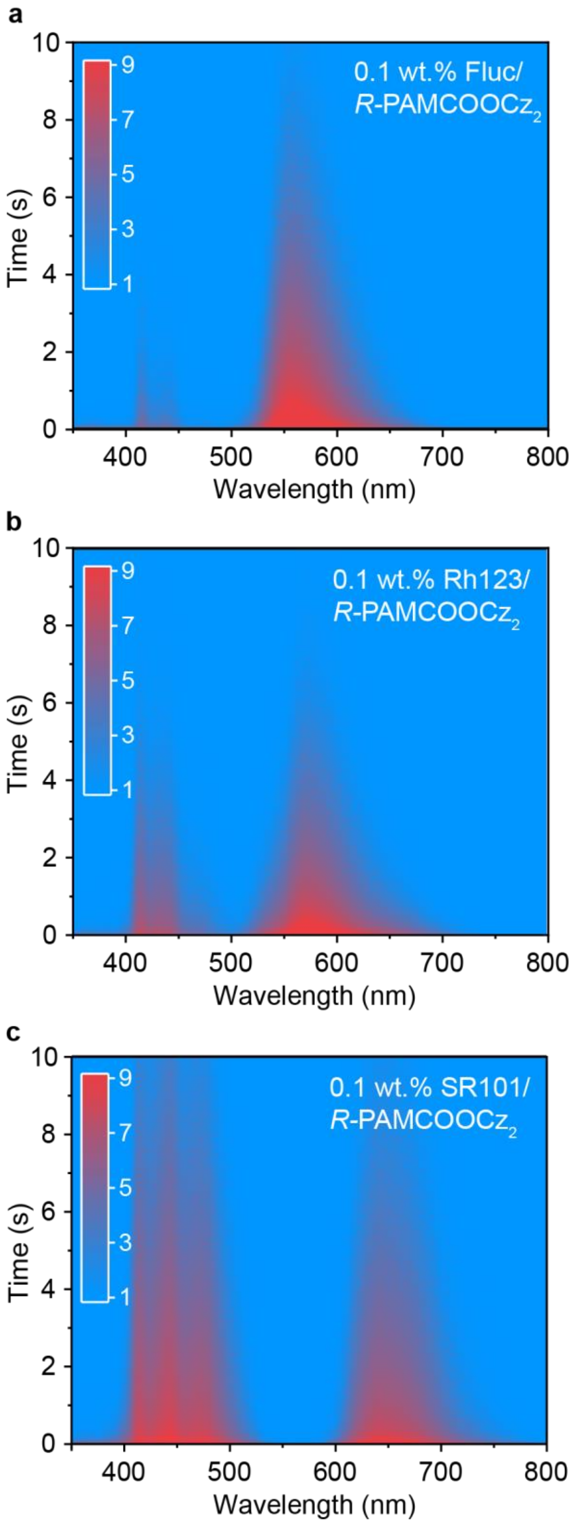

Supplementary Figure 32. TRES of (a) 0.1 wt.% Fluc/*R*-PAMCOOCz$_2$, (b) 0.1 wt.% Rh123/*R*-PAMCOOCz$_2$, and (c) 0.1wt.% SR101/*R*-PAMCOOCz$_2$ films excited by 299 nm UV light.

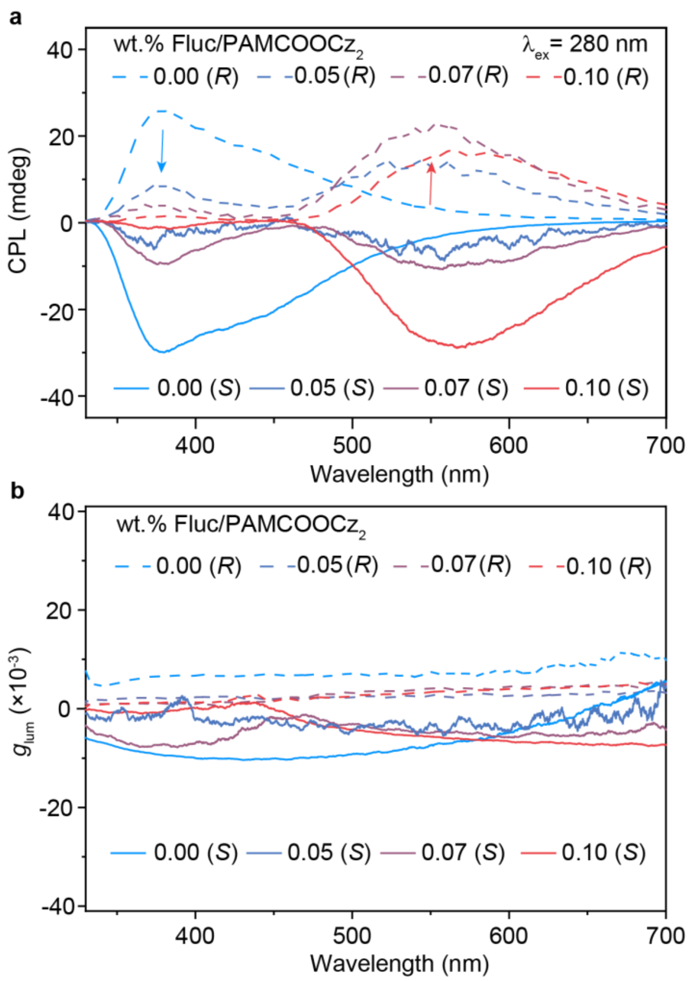

Supplementary Figure 33. (a) CPL and (b) corresponding g_{lum} values spectra of Fluc-doped *R/S*-PAMCOOCz$_2$ films at different doping weight concentrations (wt.%) excited by 280 nm UV light.

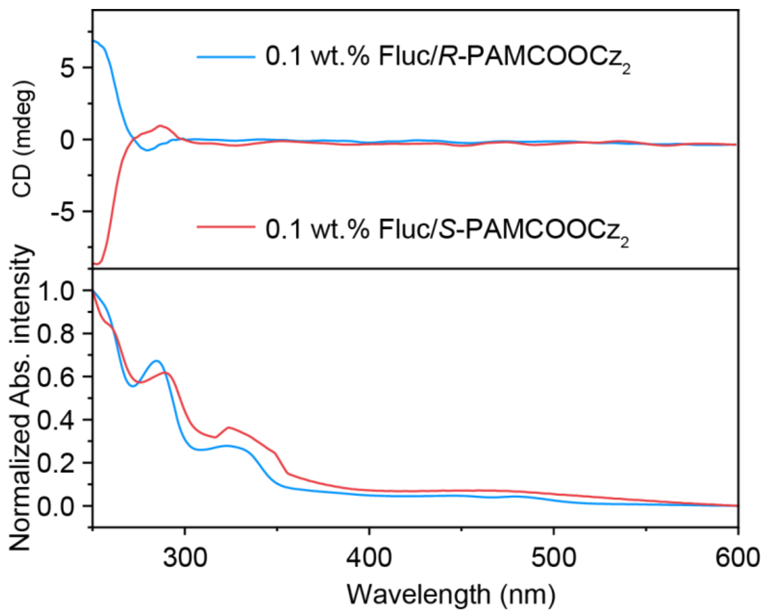

Supplementary Figure 34. CD spectra of 0.1 wt.% Fluc-doped *R/S*-PAMCOOCz$_2$ films.

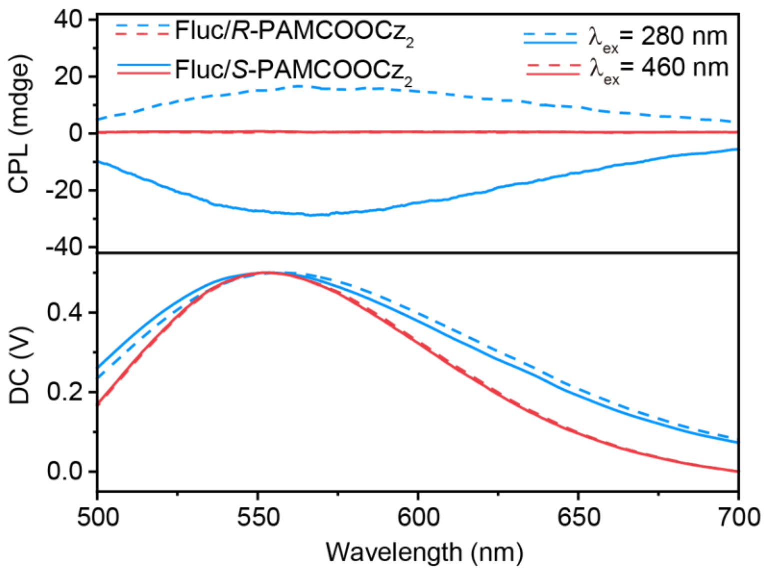

Supplementary Figure 35. The CPL properties of 0.1 wt.% Fluc/*R*-PAMCOOCz2 and 0.1 wt.% Fluc/*S*-PAMCOOCz2 films excited by 280 nm and 460 nm blue light. Noted: compared to the film excited by 280 nm, no CPL property was found in 0.1 wt.% Fluc/*R*-PAMCOOCz2 and 0.1 wt.% Fluc/*S*-PAMCOOCz2 films when the Fluc was directly excited by 460 nm, suggesting that the SACET plays a vital role in conferring the CPL afterglow nature for the fluorescent guest doped *R*/*S*-PAMCOOCz2 films.

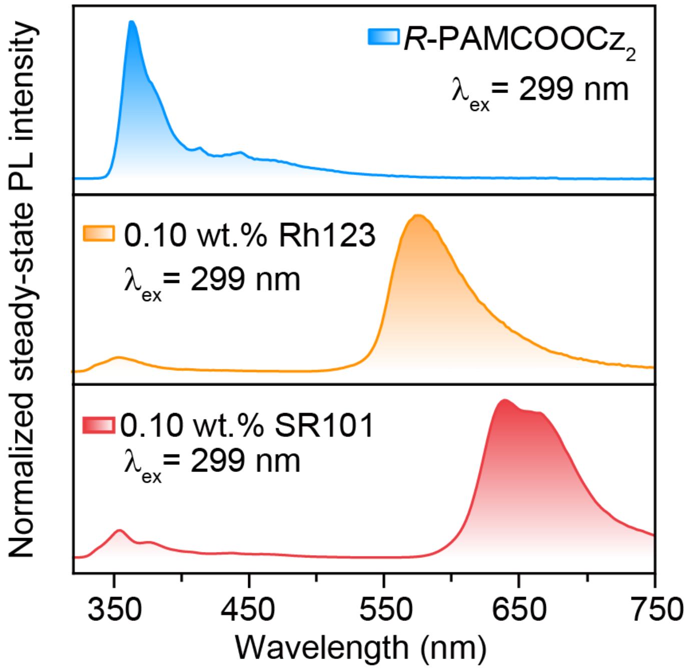

Supplementary Figure 36. Steady-state PL spectra of *R*-PAMCOOCz2, 0.10 wt.% Rh123/*R*-PAMCOOCz2 and 0.10 wt.% SR101/*R*-PAMCOOCz2 films.

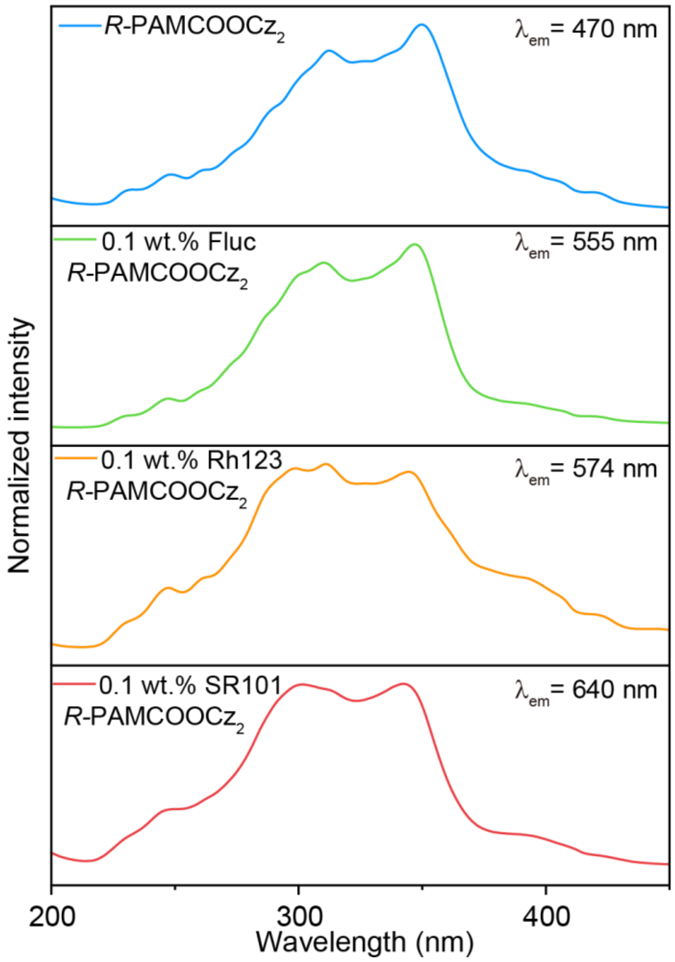

Supplementary Figure 37. Excitation-delayed PL spectra of *R*-PAMCOOCz$_2$ film as well as varied fluorescent guests doped *R*-PAMCOOCz$_2$ films.

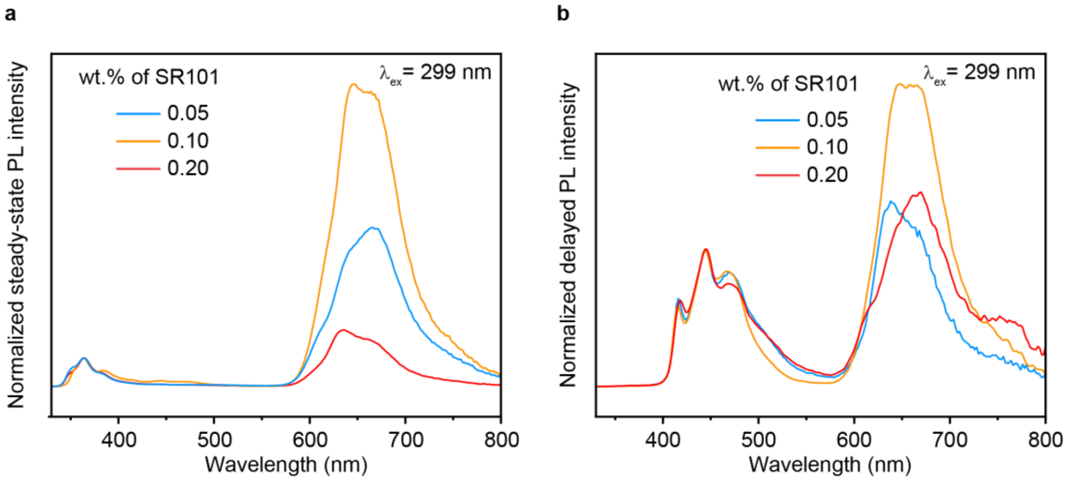

Supplementary Figure 38. (a) SSPL and (b) delayed PL (10 ms delay) spectra of SR101/*R*-PAMCOOCz$_2$ films at different doping weight concentrations (wt.%).

Supplementary Table 7. Intensity (τ_{int}) and amplitude (τ_{amp}) averaged lifetimes of *R*-PAMCOOCz$_2$, 0.10 wt.% Rh123 and SR101 of *R*/S-PAMCOOCz$_2$ films excited by 299 nm UV light.

**Samples**	λ_{phos} **(nm)**	τ_{int} **(s)**	τ_{amp} **(s)**
R-PAMCOOCz$_2$	414	3.0	2.8
0.1 wt% Rh123/*R*-PAMCOOCz$_2$	414	2.1	1.8
0.1 wt% Rh123/*R*-PAMCOOCz$_2$	574	1.9	1.3
0.1 wt% SR101/*R*-PAMCOOCz$_2$	414	2.7	2.4
0.1 wt% SR101/*R*-PAMCOOCz$_2$	645	2.2	1.8
S-PAMCOOCz$_2$	414	3.0	2.7
0.1 wt% Rh123/*S*-PAMCOOCz$_2$	414	2.3	2.0
0.1 wt% SR101/*S*-PAMCOOCz$_2$	414	2.6	2.2

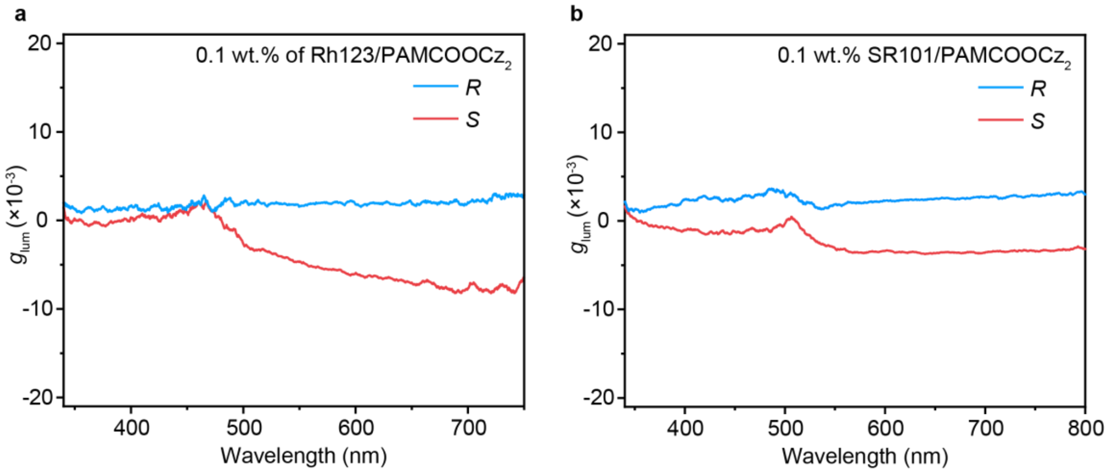

Supplementary Figure 39. g_{lum} values spectra of (a) 0.1 wt.% Rh123 doped *R*/*S*-PAMCOOCz$_2$ and (b) 0.1 wt.% SR101 doped *R*/*S*-PAMCOOCz$_2$ films.

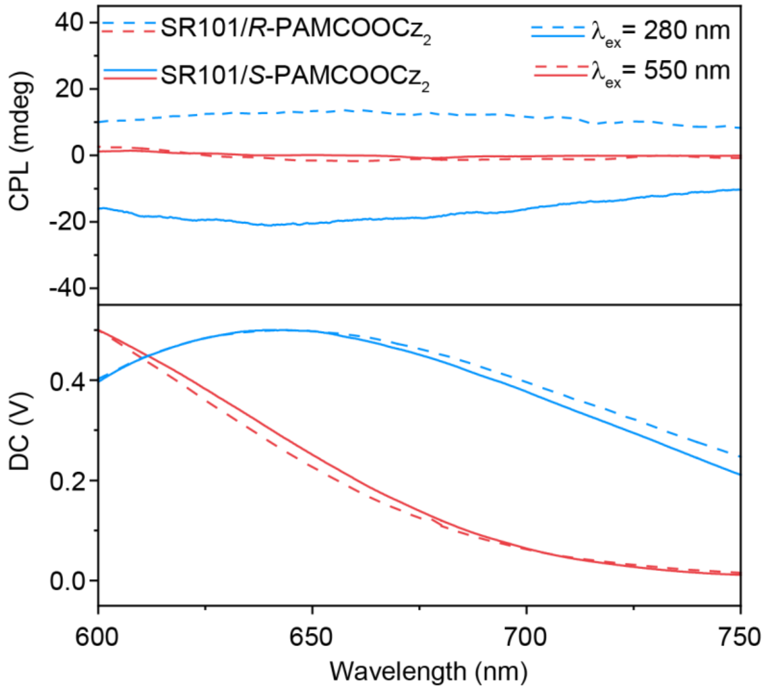

Supplementary Figure 40. The CPL properties of 0.1 wt.% SR101/*R*-PAMCOOCz$_2$ and 0.1 wt.% SR101/*S*-PAMCOOCz$_2$ films excited by 550 nm yellowish-green light. Noted: compared to the film excited by 280 nm, no CPL property was found in 0.1 wt.% SR101/*R*-PAMCOOCz$_2$ and 0.1 wt.% SR101/*S*-PAMCOOCz$_2$ films when the SR101 was directly excited by 550 nm, suggesting that the SACET plays a vital role in conferring the CPL afterglow nature for the fluorescent guest doped *R*/*S*-PAMCOOCz$_2$ films.

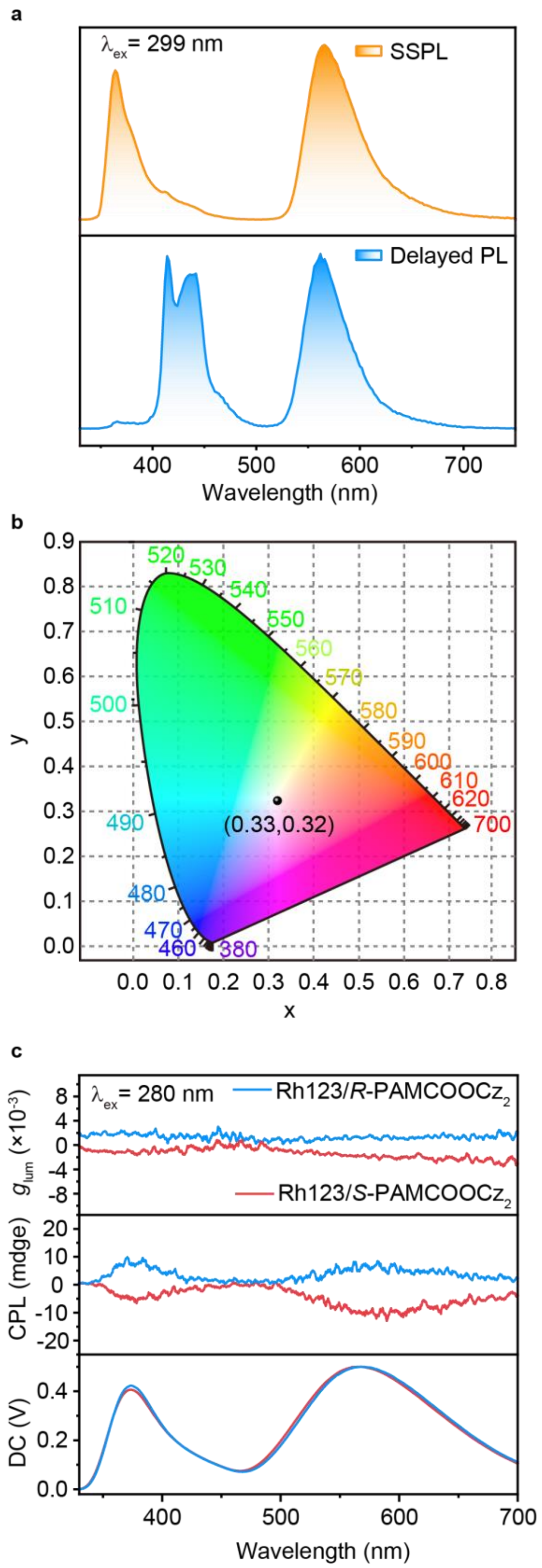

Supplementary Figure 41. (a) SSPL (top panel) and delayed PL (10 ms delay, bottom panel) spectra as well as (b) the Commission International de L'Eclairage (CIE) 1931 coordinates of 0.03 wt.% Rh123/*R*-PAMCOOCz_2 film. (c) CPL properties of 0.03 wt.% Rh123/*R*-PAMCOOCz_2 and Rh123/*S*-PAMCOOCz_2 film.

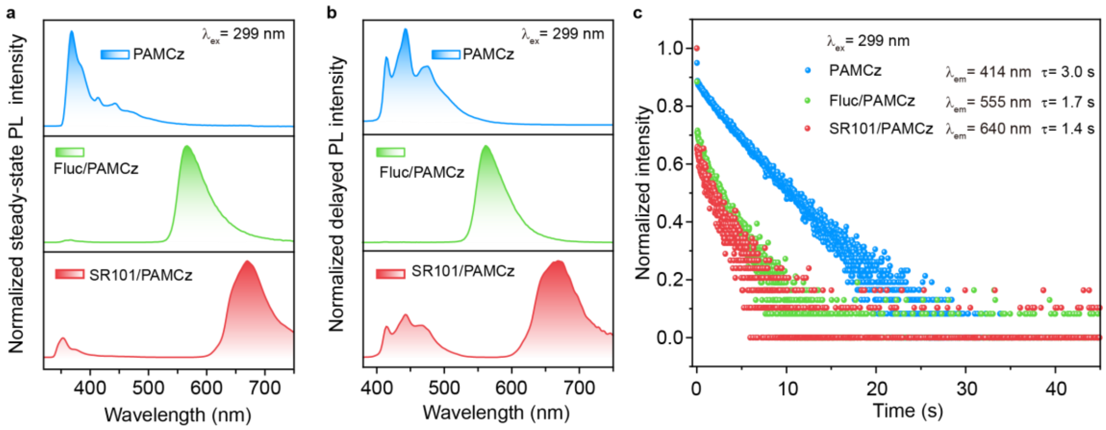

Supplementary Figure 42. (a) SSPL, (b) delayed PL (10 ms delay) and (c) afterglow lifetime decay profiles of PAMCz, Fluc/PAMCz and SR101/PAMCz films.

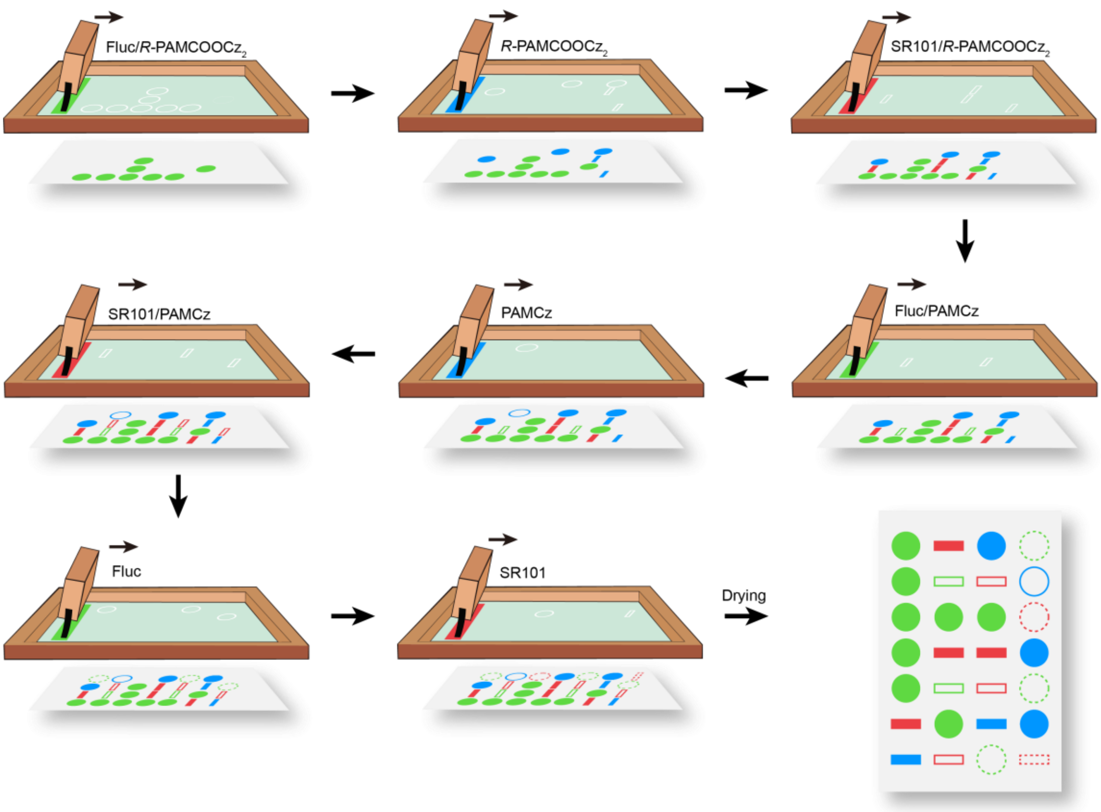

Supplementary Figure 43. Preparation of Morse code for multiplex Morse codes using screen printing technique. Detailed procedures: the prepared polymer material was firstly made into a security ink at a concentration of 1 g/mL. Secondly, the ink was printed on paper through screen printing, and different types of security inks were sequentially printed on paper. Lastly, the screen-printed multiplex Morse codes was dried in an oven at 50°C. The multiple level encryption characteristics Morse code capable of chirality, color, and ultralong lifetime could be achieved.

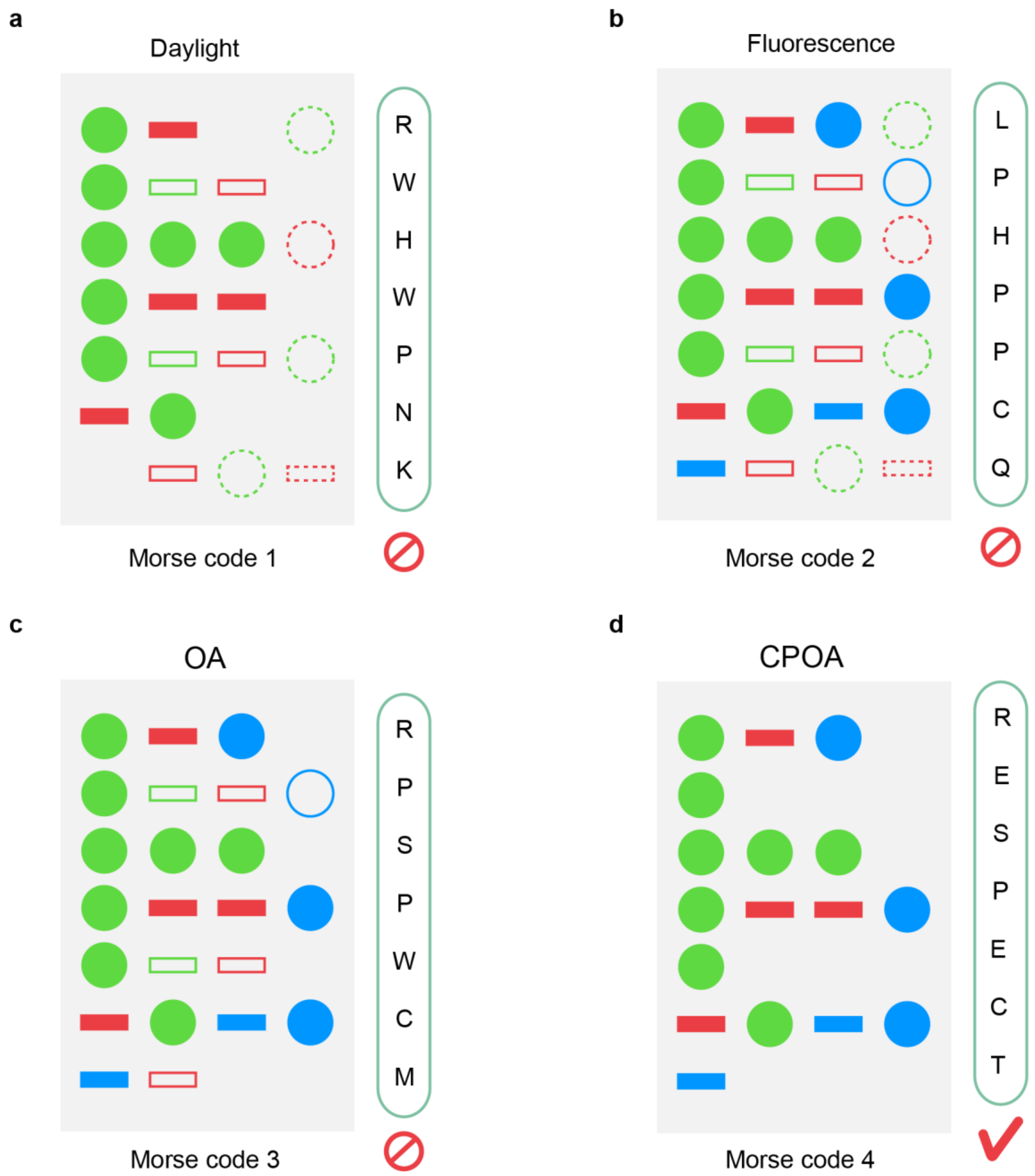

Supplementary Figure 44. Fabrication procedure of multiplex Morse Code by screen-printing and the schematic diagram corresponding photographs of varied Morse Code under daylight, under 254 nm UV light excitation and after turning off 254 nm UV light excitation.
