# Supplementary material for: Enabling robust blue circularly polarized organic afterglow through self-confining isolated chiral chromophore
Source: Nat Commun. 2024 Apr 9;15:3053. doi: 10.1038/s41467-024-47240-5 (PMC11004163; doi:10.1038/s41467-024-47240-5)
Supplement: Supplementary file 3 — Description of Additional Supplementary Files [file 41467_2024_47240_MOESM3_ESM.pdf]

Description of Additional Supplementary Files

File Name: Supplementary Data 1
Description: The atomic coordinates of the optimized ground and lowest singlet excited states of R/S-VCOOCz for ECD calculations.
