# Supplementary material for: Enabling robust blue circularly polarized organic afterglow through self-confining isolated chiral chromophore
Source: Nat Commun. 2024 Apr 9;15:3053. doi: 10.1038/s41467-024-47240-5 (PMC11004163; doi:10.1038/s41467-024-47240-5)
Supplement: Supplementary file 4 — Supplementary Data 1 [file 41467_2024_47240_MOESM4_ESM.zip › Legend of Supplementary Data 1.docx]

Legend of Supplementary Data 1: The atomic coordinates of the optimized ground and lowest singlet excited states of R/S-VCOOCz for ECD calculations.
